# The association of weight loss with changes in the gut microbiota diversity, composition, and intestinal permeability: a systematic review and meta-analysis

**DOI:** 10.1080/19490976.2021.2020068

**Published:** 2022-01-18

**Authors:** Dimitrios A Koutoukidis, Susan A Jebb, Matthew Zimmerman, Afolarin Otunla, J. Aaron Henry, Anne Ferrey, Ella Schofield, Jade Kinton, Paul Aveyard, Julian R. Marchesi

**Affiliations:** a Nuffield Department of Primary Care Health Sciences, University of Oxford, Oxford, UK; b NIHR Oxford Biomedical Research Centre, Oxford University Hospitals NHS Foundation Trust, Oxford, UK; c Department of Metabolism, Digestion and Reproduction, Imperial College London, London, UK

**Keywords:** Weight loss, microbiota, diversity, intestinal permeability, meta-analysis

## Abstract

The gut microbiome may be a mediator between obesity and health outcomes. However, it is unclear how intentional weight loss changes the gut microbiota and intestinal permeability. We aimed to systematically review and quantify this association. We searched Medline, Embase, CINAHL, Cochrane databases, and trial registries until June 2020 (PROSPERO: CRD42020205292). We included trials of weight loss interventions (energy-restricted diets, pharmacotherapy, bariatric surgery) reporting on the microbiome. Two reviewers independently completed screening, extraction, and risk assessment with the ROBINS-I tool. Pooled standardized mean differences (SMDs) were obtained from random-effects meta-analyses. Forty-seven trials with 1,916 participants (81% female) and a median follow-up of 6 months (range: 2–24) were included. Based on imprecise evidence but with fairly consistent direction of effect, weight loss was associated with a statistically significant increase in α-diversity [SMD: 0.4 (95% CI: 0.2, 0.6], *p* < .0001, I^2^ = 70%, n = 30 studies) and a statistically significant reduction in intestinal permeability [SMD: −0.7 (95% CI: −0.9, −0.4), *p* < .0001, I^2^ = 83%, n = 17 studies]. Each kg of weight loss was associated with a 0.012 (95% CI: 0.0003, 0.024, *p* = .045) increase in α-diversity and a −0.017 (95% CI: −0.034, −0.001, *p* = .038) reduction in intestinal permeability. There was clear evidence of increases in the relative abundance of *Akkermansia*, but no clear evidence of changes in individual phyla, species, or fecal short-chain fatty acids. Restricting the analyses to the studies with lower risk of bias did not materially alter the estimates. Increasing weight loss is positively associated with increases in gut microbiota α-diversity and reductions in intestinal permeability.

## Introduction

Overweight and obesity affects a quarter of the population worldwide and causes premature morbidity and mortality.^1^ Weight loss can mitigate these health risks, typically in a dose–response manner.^2,3^ The gut microbiome might be a potential mediator,^4^ as it has been hypothesized to contribute to the pathophysiology of obesity in humans, and has been an attractive target for research because it can be easily modulated by diet.^5^

Obesity, systemic inflammation, and insulin resistance are associated with lower microbiota diversity and higher intestinal permeability.^6,7^ Furthermore, dietary energy intake is negatively associated with microbiota diversity.^8^

Preclinical data support a causal link between the gut microbiome, obesity, and host metabolism including insulin resistance.^9^ Conventional mice have higher adiposity and insulin resistance than mice lacking microbiota.^10^ When transplanted to mice, the microbiota from individuals with obesity transfers the obesity-associated phenotype, including higher adiposity and systemic inflammation.^11^

However, it remains unclear how the diversity and permeability change in humans in response to weight loss and whether the changes are a generic effect of weight loss or relate to the type of intervention. Previous systematic reviews have drawn conclusions based on case reports, trials with no baseline assessment of the microbiome, a combination of human and pre-clinical trials, or focused exclusively on either bariatric surgery or specific energy-restricted diets. Some, but not all, studies indicated reductions in intestinal permeability and changes in specific phyla, such as increases in Bacteroidetes and decreases in Firmicutes, but there was no clear evidence of changes in overall microbiota diversity and none provided a quantitative estimate of change.^12–15^ Furthermore, these changes might be independent of the type of weight loss intervention (e.g., different diets, diet vs. bariatric surgery, or different types of surgery) when the same weight loss is achieved.^7,16^ This independence points toward the hypothesis that reductions in energy intake, approximated by weight loss, are the main driver of these changes.

This systematic review and meta-analysis aimed to quantitatively synthesize changes in gut microbiome and permeability following weight loss interventions, to examine consistency across interventions, and to examine whether a dose–response relationship exists.

## Methods

We conducted a prospectively registered systematic review (PROSPERO ID: CRD42020205292) following the protocol without changes. The review adhered to the PRISMA guidelines.^17^

### Eligibility criteria

We included trials of interventions to support weight loss in adults with overweight or obesity. Trials could have a single-arm, non-randomized comparative, or randomized design. Systematic reviews were screened to identify potentially eligible studies. Interventions could include energy-restricted diets, pharmacotherapy licensed for weight loss, or bariatric surgery. Exercise-only interventions or diet interventions that did not explicitly mention that the aim is weight loss or define a hypo-energetic target intake were excluded. For comparative trials, the comparator could be usual care or any weight loss intervention, as described above, of the same or different intensity. If a multi-arm trial had a single weight loss intervention arm meeting the above criteria, this specific arm was included and treated as a single-arm trial for analysis. Trial arms testing combinations of any of the above interventions with other interventions (including dietary supplements, e.g. pro-/prebiotics, or other pharmacotherapy, e.g. metformin) were excluded to eliminate confounding. The minimum accepted intervention duration for non-surgical interventions was 2 months (8 weeks) and the minimum follow-up for all trials was 2 months to allow for weight loss to occur. There were no restrictions on maximum duration and follow-up.

Studies had to report at least one of the following outcomes to be included: (a) α-diversity (e.g., Shannon index, Chao1 index, observed operational taxonomic units (OTUs), Simpson index, phylogenetic diversity); (b) β-diversity (e.g., Bray-Curtis and UniFrac distances), (c) changes in relative abundance of lineages at various phylogenetic resolutions (i.e. phylum, genus, species); (d) intestinal inflammation (fecal calprotectin, α-1-antitrypsin); (e) intestinal permeability (fecal zonulin, plasma/serum zonulin, lipopolysaccharide, lipopolysaccharide binding protein, lactulose/mannitol/(sucrose) test,^18 (51)^Cr-Ethylenediaminetetraacetic acid (EDTA)); (f) fecal short-chain fatty acids (acetate, propionate, and butyrate). Studies had to report estimates of effect and variance in each of these outcomes (or provide data to allow for their calculation) in an intervention cohort of at least 10 people, so that we could reasonably pool data in a meta-analysis.

### Search strategy and information sources

An experienced librarian created the search strategy (which was published with the review protocol and is available in the supplementary material) and searched the MEDLINE, Embase, CINAHL, and Cochrane databases and two trial registries from inception to June 2020. In October 2021, we ran an updated search of follow-up publications for trial protocols identified in the original search. There were no language restrictions.

### Study selection and data collection

A reviewer (one of DAK, MZ, AO, JAH, AF, ES, JK) screened titles and abstracts and, among the seven of us, two at a time paired up to independently screen the full texts with an online standardized tool.^19^ Two of us at a time also independently extracted the data pre-specified in the protocol using a pre-defined and pre-piloted form and assessed the risk of bias due to confounding, selection of participants, classification of interventions, deviations from the intended intervention, missing data, measurement of outcomes, and selective reporting as low, moderate, serious, or critical using the ROBINS-I tool.^20^ Full-text screening, data extraction, and risk of bias assessment were completed in duplicate for each study and conflicts were resolved through discussion or referral to a third reviewer (DAK or PA). We did not contact study authors for additional information.

### Synthesis

A random effects meta-analyses between pre- and post-exposure using the DerSimonian and Laird method was conducted.^21^ Given the substantial variations in the method of analysis and reporting of gut microbiota diversity markers, data were analyzed using standardized mean differences [95% confidence intervals (CIs)] as effect estimates. A pre-specified subgroup analyses (a) by type of intervention (i.e. diet vs. pharmacotherapy vs. bariatric surgery) and (b) between different types of surgery and different types of diet (food-based diet advice vs. provided formula-based diet), where data allowed, was also performed. Aiming to reduce bias due to selective reporting in genera and species, we restricted the analyses to only genera and species reported in at least four trial groups. Following peer-review, we conducted (a) a sensitivity analysis per individual permeability marker and (b) a sensitivity analysis by the analytical method for *α-*diversity and, where data allowed, for phyla and genera. Statistical heterogeneity was assessed with the I^2^ statistic, and exploratory meta-regressions between weight change and changes in α-diversity and permeability were performed, to examine whether weight loss was a significant mediator.

Each domain of risk of bias was scored as 1, 2, 3, or 4, if they were judged as at low, moderate, serious, or critical risk of bias, respectively. To examine whether risk of bias affected the estimates, we run a sensitivity analysis including only studies that had both a score ≤11 (median score) and no domain scored as 4 (i.e., critical). We evaluated the consistency and precision of the evidence. Consistency was based on direction of effect and analyses were judged consistent if all studies followed the same direction. Precision was based on the confidence intervals around the point estimates. Additionally, among studies with consistent direction, precision was evaluated based on whether confidence intervals were crossing zero. Analyses were judged as clear and convincing when the direction of effect was the same in all studies, limited statistical heterogeneity was present, and confidence intervals were both tight around the point estimate and not crossing zero. Publication bias was examined through visual inspection of funnel plots. Analyses were conducted using the package “meta” in R v4.0.3.

## Results

**Figure 1.**
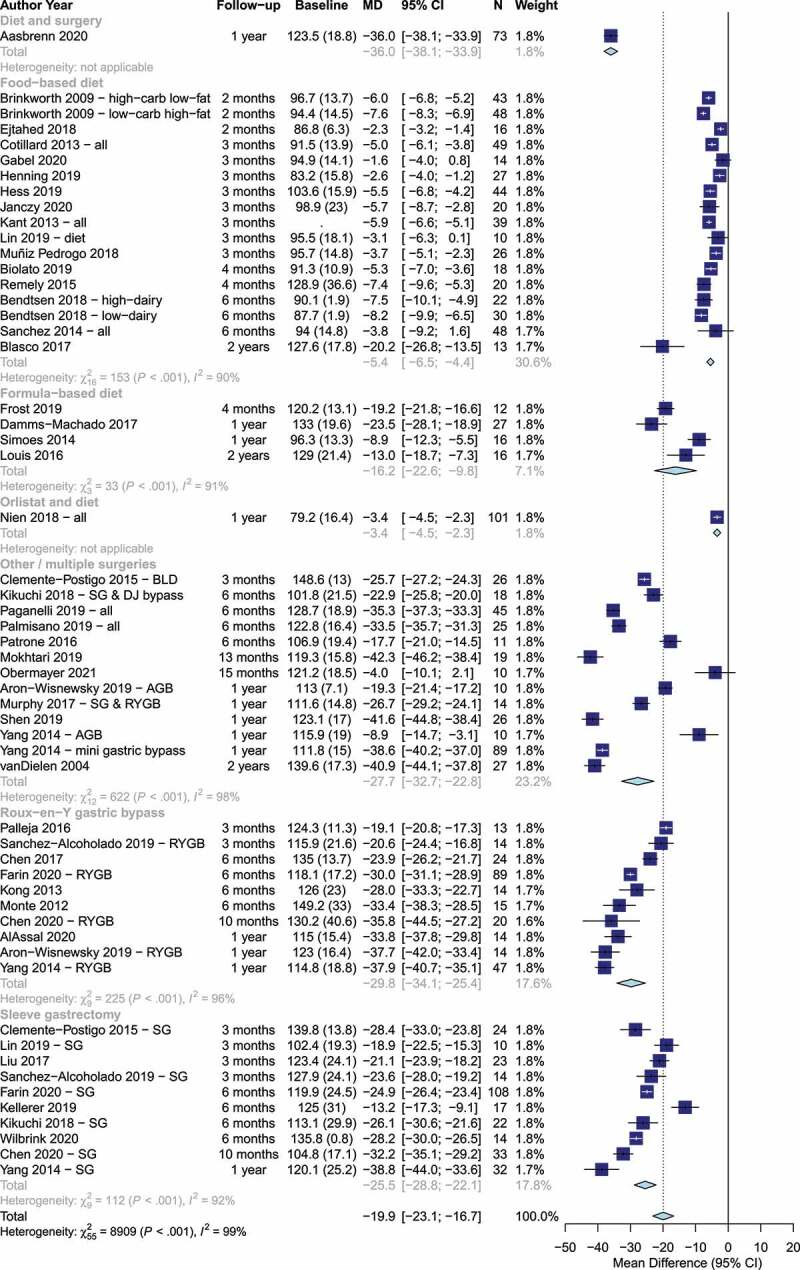
Weight loss by intervention type and length of follow-up.

The search returned 2,717 results. Of those, 188 were screened at full-text stage and 47 trials were included (PRISMA flowchart in Supplementary Figure 1) with 1,916 participants. Participants were 81% female with mean (SD) age: 42 (12) years). Forty trials were in high-income countries with the remaining in upper-middle-income countries.^22–28^ Nine trials were in Asia,^7,25,27,29–31^ two in Oceania^,32,33^ one in South America,^22^,and the rest in Europe (*n*=27), North America (*n*=7) or a combination (*n*=1).^34^ Twenty-five trials examined forms of bariatric surgery,^22,26,28,29,34–50^ fifteen trials examined dietary interventions advising on hypo-energetic diets,^18,24,51–61^ four trials examined formula-based hypo-energetic diets,^62–64,65^ two trials examined behavioral support programmes and bariatric surgery,^30,66,67^ and one trial examined orlistat and behavioral support (Table 1).^31^ The median follow-up was 6 months (range: 2–24, interquartile range: 3–12). The average weight loss was −19.9 kg (95% CI: −23.1, −16.7) and was significantly different among interventions (food-based diet: −5.4 kg, formula-based diet: −16.2 kg, sleeve gastrectomy: −25.5 kg, Roux-en-Y gastric bypass: −29.8 kg, *p* < .001, Figure 1).

**Table 1.** Characteristics of included trials

Trial, country	N (N female)	Age	N, T2D	Intervention	Follow-up (months)
Louis 2016, Germany^62^	16 (9)	40 (8)	NR	FD	24
Damms-Machado 2017, Germany^63^	27 (14)	44 (8)	0	FD	12
Simoes 2014, Finland^65^	16 (16)	NR	NR	FD	12
Frost 2019, Germany^64^	12 (8)	57 (7)	12	FD	4
Blasco 2017, Spain^51^	17 (9)	54 (7)	0	DA	24
Sanchez 2014, Canada^18^	63 (39)	37 (10)	0	DA	6
Bendtsen 2018, Denmark^52^	40 (35)	40 (6)	0	DA	6
	40 (34)	45 (13)	0	DA	6
Remely 2015, Austria^53^	33 (NR)	43 (14)	0	DA	4
Biolato 2019, Italy^54^	20 (2)	43 (NR)	0	DA	4
Hess 2019, Denmark^55^	44 (25)	48 (9)	NR	DA	3
Cotillard 2013, France^56^	49 (41)	NR	0	DA	3
Medina-Vera 2019, Mexico^23^	25 (13)	50 (11)	25	DA	3
Kant 2013, UK^57^	39 (NR)	44 (12)	NR	DA	3
Henning 2019, USA^58^	27 (20)	36 (11)	NR	DA	3
Muñiz Pedrogo 2018, USA^59^	26 (21)	54 (8)	NR	DA	3
Janczy 2020, Poland^60^	20 (15)	37 (14)	NR	DA	3
Lin 2019, Taiwan^30^	10 (6)	38 (11)	0	DA	3
	10 (6)	36 (10)	0	S: SG	3
Gabel 2020, USA^61^	14 (0)	NR	0	DA	3
Ejtahed 2018, Iran^24^	22 (16)	34 (7)	0	DA	2
Brinkworth 2009, Australia^32^	43 (25)	51 (8)	NR	DA	2
	48 (30)	50 (8)	NR	DA	2
Aasbrenn 2020, Norway^66,67^	143 (110)	43 (9)	NR	DA & RYGB/SG	12
Nien 2018, Taiwan^31^	101 (60)	49 (10)	NR	Orlistat/DA	12
van Dielen 2004, Netherlands^35^	27 (22)	38 (8)	NR	S: VGB/AGB	24
Obermayer 2021, Austria^50^	10 (6)	48 (9)	10	S: Endobarrier^TM^	15
Mokhtari 2019, Iran^25^	23 (23)	37 (11)	38	S: OAGB	13
Yang 2014, Taiwan^7^	10 (5)	35 (12)	NR	S: AGB	12
	89 (68)	32 (10)	NR	S: MGB	12
	47 (35)	33 (9)	NR	S: RYGB	12
	32 (13)	34 (9)	NR	S: SG	12
Al Assal 2020, Brazil^22^	25 (25)	46 (8)	25	S: RYGB	12
Aron-Wisnewsky 2019 AGB, France^36^	10 (10)	36 (8)	0	S: AGB	12
	14 (14)	42 (8)	15	S: RYGB	12
Shen 2019, USA, Spain^37^	26 (NR)	NR	8	S: RYGB/SG	12
Murphy 2017, New Zealand^33^	14 (NR)	NR	6	S: RYGB/SG	12
Chen 2020, China^28^	33 (19)	33 (10)	21	S: RYGB	10
	54 (41)	30 (8)	12	S: SG	10
Chen 2017, China^27^	24 (10)	52 (10)	24	S: RYGB	6
Farin 2020, France, USA, UK^34^	89 (73)	45 (11)	32	S: RYGB	6
	108 (75)	42 (15)	21	S: SG	6
Kellerer 2019, Germany^38^	17 (NR)	42 (9)	6	S: SG	6
Monte 2012, USA^39^	15 (11)	45 (9)	15	S: RYGB	6
Palmisano 2019, Italy^40^	25 (21)	45 (9)	8	S: RYGB/SG	6
Patrone 2016, Italy^41^	11 (11)	51 (NR)	11	S: BIB	6
Wilbrink 2020, Netherlands^42^	14 (NR)	NR	NR	S: SG	6
Paganelli 2019, Netherlands^43^	45 (36)	43 (NR)	4	S: RYGB/SG	6
Kikuchi 2018, Japan^29^	22 (22)	41 (9)	11	S: SG	6
	18 (8)	48 (11)	16	S: SG & DJB	6
Kong 2013, France^44^	30 (30)	NR	7	S: RYGB	6
Campisciano 2018, Italy^45^	10 (NR)	NR	NR	S: RYGB	3
	10 (NR)	NR	NR	S: SG	3
Clemente-Postigo 2015, Spain^46^	26 (NR)	43 (7)	11	S: BPD	3
	24 (NR)	43 (11)	7	S: SG	3
Liu 2017, China^26^	23 (16)	29 (8)	NR	S: SG	3
Palleja 2016, Denmark^47^	13 (8)	NR	7	S: RYGB	3
Sanchez-Alcoholado 2019, Spain^48^	14 (10)	NR	NR	S: RYGB	3
	14 (10)	NR	NR	S: SG	3
Troseid 2013, Norway^49^	49 (34)	43 (9)	16	S: RYGB/DS	3

AGB: Adjustable gastric banding, BIB: Bilio-intestinal bypass, BPD: Bilio-pancreatic diversion, DA: Hypo-energetic dietary advice, DJB: Duodenojejunal bypass, DS: Duodenal switch, FD: Formula-based diet, RYGB: Roux-en-Y gastric bypass,

S: Surgery, SG: Sleeve gastrectomy, T2D: Type 2 diabetes, VBG: vertical banded gastroplasty. Age as mean ± SD.

### Gut microbiota α-diversity changes associated with weight loss

**Figure 2.**
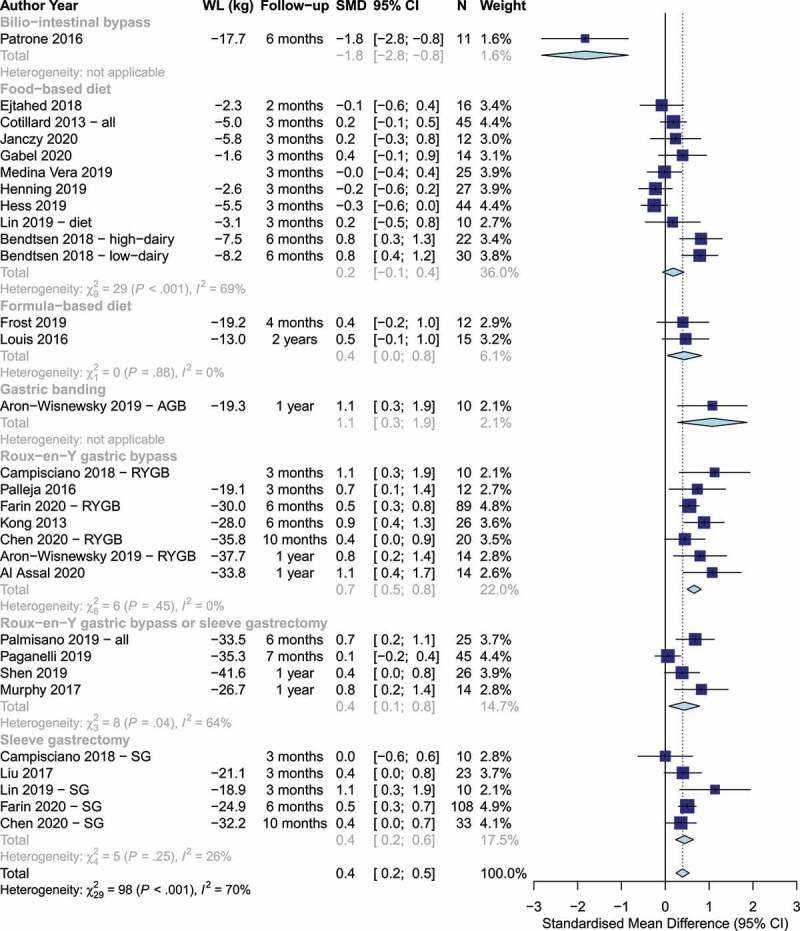
Changes in α-diversity by weight loss intervention. Positive and negative values indicate increases and decreases in α-diversity, respectively. (WL: Weight loss, SMD: Standardized mean difference).

All studies used fecal samples for analyses. Overall, weight loss was associated with a statistically significant increase in α-diversity based on imprecise evidence with fairly consistent direction of effect [SMD: 0.4 (95% CI: 0.2, 0.6], *p* < .0001, I^2^= 70%, n = 30 studies, Figure 2). The evidence of this association was clear and consistent for Roux-en-Y gastric bypass [SMD: 0.7 (95% CI: 0.5, 0.8), I-squared= 0%, n = 7 studies]. The evidence was imprecise, but mostly consistent for sleeve gastrectomy [SMD: 0.4 (95% CI: 0.2, 0.6), I^2^= 26%, n = 5 studies] and for mixture of Roux-en-Y gastric bypass and sleeve gastrectomy [SMD: 0.4 (95% CI: 0.1, 0.8), I^2^= 64%, n = 4 studies]. The evidence was imprecise and inconsistent for food-based dietary weight loss advice with no statistically significant change in the pooled α-diversity estimate (SMD: 0.2 (95% CI: −0.1, 0.4), I^2^= 69%, n = 10 studies). In sensitivity analysis, the majority of the heterogeneity among food-based trials was explained by the two groups of one study^52^ that achieved the largest weight loss (7.5–8.2 kg), largest increase in α-diversity (SMD.. 0.8), and longest follow-up (6 months), as excluding this study led to a revised total estimate among food-based trials (SMD 0.0 (95% CI: −0.2, 0.2), I^2^= 19%, n = 8). The formula-based diets indicated an imprecise, but consistent effect (SMD: 0.4 (95% CI: 0.04, 0.8), I^2^= 0%, n = 2 studies). Individual analysis of α-diversity markers showed statistically significant improvements in the Shannon index, the OTUs count, the abundance-based coverage estimator (ACE) index, and gene richness, but no evidence of a change in the Simpson index, the phylogenetic diversity, and the Chao1 index (Figures S2-S8). There was no evidence of difference in estimates by the analytical method used (Figure S9).

### Changes at the taxonomic level of phylum associated with weight loss

There was no clear evidence of change in the phyla reported (Figure S10). There was inconsistent and imprecise evidence that, following weight loss, Firmicutes had lower abundance, the Firmicutes/Bacteroidetes ratio was lower, and Proteobacteria and Verrucomicrobia had higher abundance, but these changes were not significant. In sensitivity analysis, there was no evidence that estimates differed by sequencing method for Firmicutes, Bacteroidetes, and Verrucomicrobia. There was suggestive evidence that changes in Actinobacteria and Proteobacteria were dependent on the sequencing method used, but these results were driven by subgroups of a single study (Figures S11-S15).

### Changes at the taxonomic level of genus associated with weight loss

There was clear evidence of increases in the relative abundance of *Akkermansia* [SMD: 0.5 (95% CI: 0.3, 0.7), I^2^=0%, n = 4 studies]. There was consistent, but imprecise evidence of increases in abundance of *Bacteroides* [SMD: 0.3 (95% CI: 0.1, 0.6), I^2^=0%, n = 5 studies] and fairly consistent, but imprecise evidence of reductions in *Bifidobacterium* [SMD: −0.5 (95% CI: −1.0, 0.0), I^2^=93%, n = 11 studies]. There was no clear evidence of changes in the abundance of *Butyricimonas, Granulicatella, Lactobacillus* except some indicative trends (Figure S16). In sensitivity analysis, there was no evidence that the estimates for *Bifidobacterium* and *Lactobacillus* differed by analytical method used (Figures S17-S18).

### Changes at the taxonomic level of species associated with weight loss

There were no sufficient data to allow meta-analysis at the species level.

### Gut microbiota β-diversity

We were unable to conduct a meta-analysis of changes in β-diversity because trials typically reported beta diversity in graphical format without data formatted appropriately for meta-analysis.

### Fecal short-chain fatty acids changes associated with weight loss

There was no evidence of change in fecal acetate [−5.3 mmol/l (95% CI: −14.2, 3.7), *p* = .25, I^2^=89%], butyrate [−0.4 mmol/l (95% CI: −2.9, 2.1), *p* = .78, I^2^= 68%], propionate [−2.0 mmol/l (95% CI: −4.8, 0.8), *p* = .16, I^2^= 83%], or total short-chain fatty acids [−6.5 mmol/l (95% CI: −13.4, 0.4), *p* = .066, I^2^= 40%] based on 4 trials (Figure S19).

### Intestinal inflammation and permeability changes associated with weight loss

**Figure 3.**
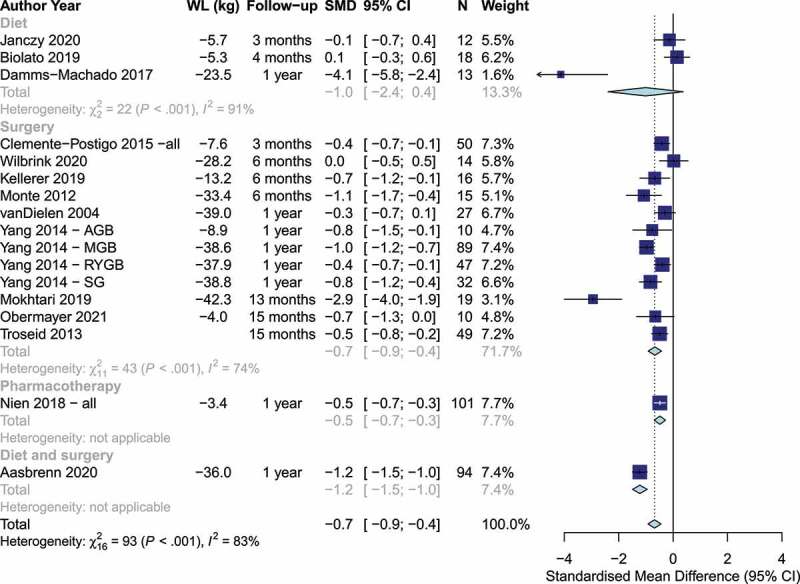
Changes in markers of intestinal permeability by weight loss intervention. Positive and negative values indicate increases and decreases in intestinal permeability, respectively. (WL: Weight loss, SMD: Standardized mean difference).

Studies measured intestinal inflammation with a variety of markers, including fecal zonulin,^60,63^ plasma or serum zonulin,^18,38,66^ 51Cr-EDTA,^54^ lipopolysaccharide,^23,39,46,49^ lipopolysaccharide binding protein,^7,25,31,35,38,46^ and markers of the lactulose/mannitol/(sucrose) test (Table S1).^38,42,63^ Weight loss was associated with a statistically significant reduction in intestinal permeability [SMD: −0.7 (95% CI: −0.9, −0.4), *p* < .0001, I^2^= 83%, n = 17 studies], with no significant differences between dietary and bariatric surgery trials (*p* = .64, Figure 3). However, the evidence was more consistent and precise among trials of bariatric surgery than among dietary interventions. In sensitivity analysis, there was evidence that the subgroup estimate of the diet trials was driven by the dietary trial with the largest weight loss (−23.5 kg) and largest change in intestinal permeability (SMD: 4.1), as there was no evidence of an effect or of heterogeneity [SMD: 0.03 (95% CI: −0.33, 0.39), I^2^=0%, n = 2 studies] among the remaining 2 diet trials with weight loss between −5.3 kg and −5.7 kg. The variety of bariatric procedures employed in trials assessing intestinal permeability precluded an analysis by surgery type. Additionally, due to limited data, we were able to conduct sensitivity analysis only for lipopolysaccharide binding protein, lipopolysaccharide, and lactulose:mannitol ratio, which showed results broadly consistent with the main analysis (Figures S20-S22). There was no evidence of change in fecal calprotectin, a marker of intestinal inflammation, based on two trials [−5.9 mcg/g (95% CI: −43.2, 31.5), *p* = .76, I^2^= 93%] (Figure S23).

### Risk of bias within and across studies

Risk of bias varied within studies (Table S2). Ten and three studies scored low and critical on risk of bias for confounding, respectively, 22 and 11 scored low and critical on risk of bias for missing data, and 12 and 14 scored low and critical on risk of bias for selective reporting with the remaining studies scoring at moderate or serious risk of bias. Restricting the α-diversity and permeability analyses to studies that were judged overall at lower risk of bias did not materially alter the findings [α-diversity SMD: 0.5 (95% CI: 0.2, 0.7), I^2^= 53%, n = 7 studies, and permeability SMD: −0.6 (95% CI: −0.9, −0.4), I^2^= 83%, n = 11 studies] (Figures S24-S25).

On visual inspection, there was no evidence of asymmetry in the funnel plot of α-diversity (Figure S26A) and excluding the study^41^ at the bottom left did not materially change the results [SMD: 0.4 (95% CI: 0.3; 0.6), *p* < .0001, I^2^= 66%, n = 29 studies]. There was evidence of asymmetry in the funnel plot of intestinal permeability (Figure S26B). Excluding the three studies ^25,63,66^ at the left side of the plot attenuated the effect estimate and reduced heterogeneity [SMD: −0.5 (95% CI: −0.7, −0.3), *p* < .0001, I^2^= 63%, n = 14 studies]. This exclusion did not change the interpretation of the overall estimate or the estimate in the surgical trials but led to no evidence of effect among diet trials (Figure S27).

### Meta-regression examining a dose–response relationship between weight loss and changes in α-diversity and intestinal permeability

**Figure 4.**
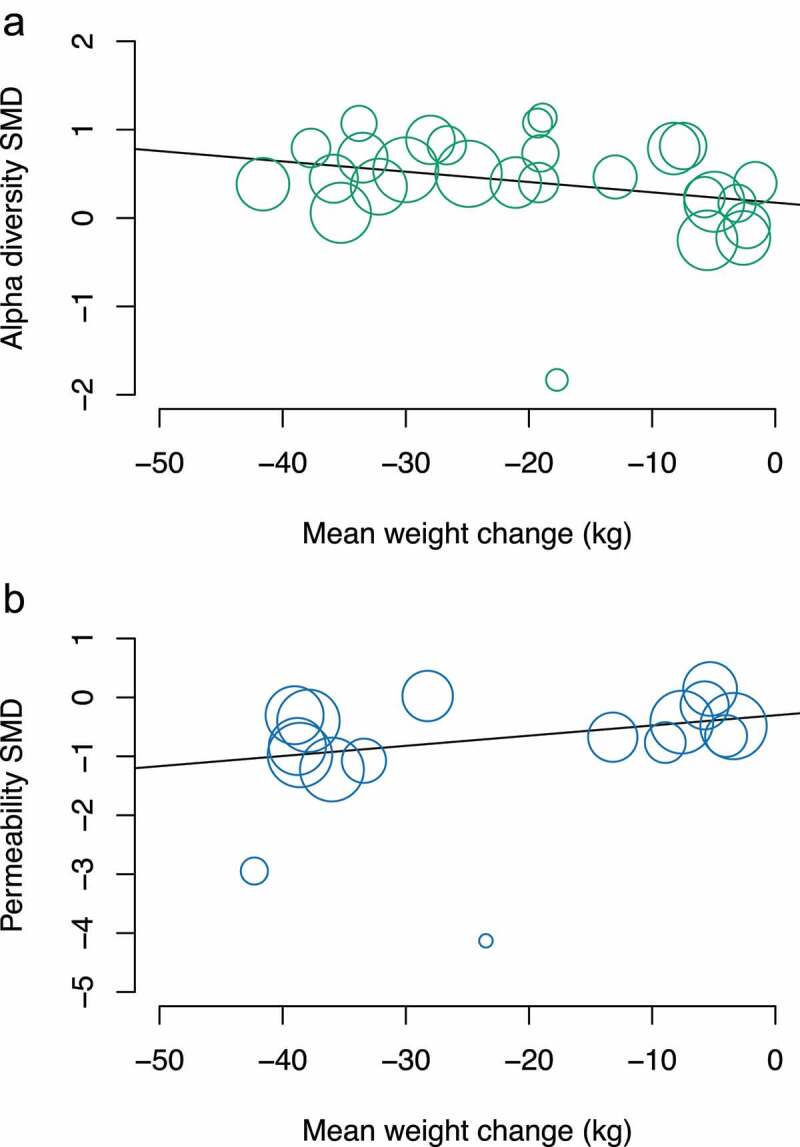
Meta-regression of change in weight and change in (a) α-diversity and (b) intestinal permeability markers.

In meta-regression where sufficient data were available, each kg of weight loss was associated with a 0.012 (95% CI: 0.0003, 0.024, *p* = .045) increase in the SMD of α-diversity markers. Furthermore, every kg of weight loss was associated with a −0.017 (95% CI: −0.034, −0.001, *p* = .038) change (reduction) in the SMD of intestinal permeability (Figure 4).

## Discussion

In people with overweight and obesity, weight loss interventions were associated with significant improvements in the α-diversity of the gut microbiota and in intestinal permeability. These associations followed a dose–response pattern. However, there was no evidence of change in intestinal inflammation, short-chain fatty acids, individual phyla, genera, and species except increases in *Akkermansia* and *Bacteroides* and decreases in *Bifidobacterium*. Restricting the analyses to studies with lower risk of bias did not materially alter the estimates.

Using data from intervention trials from 17 countries, our study provides the first quantitative estimate of the magnitude of the association between changes in weight and expected changes in microbiota and intestinal permeability. Our results are consistent with systematic qualitative syntheses of the evidence on intestinal permeability.^15^ Previous systematic reviews have not drawn firm conclusions on changes in α-diversity^12–14^ and our review is the first to show consistent evidence of increases in α-diversity. In previous reviews, changes in individual phyla, genera, and species have been reported qualitatively, shown to be inconsistent, and are likely to have a high risk of bias, because some of those reported changes are based on very few studies (typically less than 3), with small sample sizes, sometimes with short duration, and sometimes based on pooled data from both humans and animals.^12–15^ We aimed to minimize such biases by pooling data for individual phyla, genera, and species only if they were reported in at least 4 studies. Additionally, we pre-specified confounding variables, a minimum sample size, and a minimum length of follow-up of 2 months, since very short-term dietary modulation of the gut microbiome has been followed by recovery to the original microbiota profile.^68^ Given the limited number of randomized controlled trials examining our research question, we included any trial design in humans and we followed the Cochrane methods to minimize bias. We also excluded combinations of weight loss interventions with other interventions that may confound the observed effects (e.g., probiotics have potent effects on the microbiota and are associated with small reductions in body weight).^69,70^ There was substantial variability in the methods used to assess and report on the gut microbiota and there is no gold-standard method. To reduce biases by assessment method, we pooled standardized mean differences from multiple outcomes. The field would benefit from standardized pre-specified analysis and reporting as well as detailed reporting of inclusion and exclusion criteria.^71^

The high statistical heterogeneity is a limitation, but we hypothesized that the weight loss is the main driver of changes and, therefore, we pooled data from interventions of multiple types, intensity, and follow-up. This hypothesis is supported by the fact that heterogeneity was markedly reduced in the α-diversity analysis to between 0% and 26% in the subgroups of Roux-en-Y gastric bypass, sleeve gastrectomy, and formula-based diet and the α-diversity estimates in each subgroup followed a dose–response pattern in line with the different amount of weight loss in each of these subgroups. Regarding the food-based diet, the heterogeneity reduced from 69% to 19% after excluding the two groups from a single study that had the largest weight loss, largest increase in α-diversity, and longest follow-up. The case was similar for the intestinal permeability analysis among the diet trials. Furthermore, there was no strong evidence of an independent effect of length of follow-up. Together with the meta-regression results, these observations support the dose–response effect of weight loss on microbiota and intestinal permeability and point toward the suggestion that >5% weight loss is necessary to observe significant changes.

We aimed to study the effects of weight loss interventions on the microbiota so excluded *a priori* dietary trials without energy restriction. Most studies we included that aimed to enact dietary change advised a “healthy eating” energy-restricted diet, and the very modest differences between interventions in the macronutrient composition are unlikely to have greatly influenced the microbiome in this context.^8,72^ Although the formula-based diets would lead to very different dietary intake in the first 3–4 months of the formula-based period, most studies reported on microbiome changes long after participants had transitioned to a “healthy eating” diet (at 1–2 years).

Despite the substantial inter-individual variability reported in the gut microbiome,^73^ our review shows that weight loss in people with overweight and obesity can consistently lead to changes in the direction of a microbiome profile that is typically seen in individuals with healthy weight, such as higher α-diversity and lower intestinal permeability.^6,7^ The current review lends support to the hypothesis that reductions in energy intake, approximately measured by weight loss, increase gut microbiota α-diversity and reduce bacterial metabolites, such as lipopolysaccharide. These alterations may subsequently lead to higher tight junction cohesion, lower intestinal permeability, lower exposure of the liver to these metabolites and inhibition of pro-inflammatory pathways. Larger adequately powered randomized controlled trials with long-term follow-up are needed to clearly establish causality.

The exact changes at phylum, genus, and species level that lead to higher α-diversity require further investigation. The evidence of overall diversity changes but lack of evidence on changes on most phyla, genera, and species might be explained by underreporting of detailed microbiota changes (i.e., reporting of only overall diversity changes), since the majority of the pooled estimates at these levels were based only on a few studies. Despite this lack of evidence, there was clear evidence that weight loss increased the abundance of the genus *Akkermansia*. This increase is in line with both observational data of lower abundance of *Akkermansia muciniphila* in people with overweight and obesity and a double-blind proof-of-concept trial indicating that *A. muciniphila* supplementation may lead to larger weight loss and improvements in liver and cardio-metabolic biomarkers.^74^ However, other pilot trials of oral fecal microbiota transplantation for the treatment of obesity do not show changes in weight.^75,76^ Whether improving the gut microbiome profile directly leads to larger weight loss requires further research. Given the modest effect size seen in this review and the complex mechanism of energy homeostasis, it is plausible that the direct effect of the microbiome in weight is modest and, thus, large studies are needed to observe a meaningful effect.

Fecal short-chain fatty acids are implicated in the regulation of appetite, energy intake, and glucose by promoting the release of appetite-reducing gut hormones.^77^ Furthermore, delivery of propionate to the human colon prevents weight gain,^78^ but we found no evidence that weight loss was directly associated with changes in fecal short-chain fatty acids. Additionally, weight loss interventions may affect other bacterial metabolites beyond those examined here^26^ and future reviews of serum and urine metabolome warrant consideration.

In conclusion, weight loss is associated in a dose–response manner with increases in gut microbiota α-diversity and reductions in intestinal permeability.

## Supplementary Material

Supplemental Material

## Data Availability

The authors confirm that the data supporting the findings of this study are available within the article, its supplementary materials, and the referenced publications from which the data were extracted.
