# Supplementary material for: The association of weight loss with changes in the gut microbiota diversity, composition, and intestinal permeability: a systematic review and meta-analysis
Source: Gut Microbes. 2022 Jan 18;14(1):2020068. doi: 10.1080/19490976.2021.2020068 (PMC8796717; doi:10.1080/19490976.2021.2020068)
Supplement: Supplemental Material [file KGMI_A_2020068_SM1647.zip › supplementary/supplementary data.pdf]

Supplementary material

Koutoukidis et al. The association between weight loss with changes in the gut microbiota diversity, composition, and intestinal permeability: a systematic review and meta-analysis

Figure S1: PRISMA Flowchart

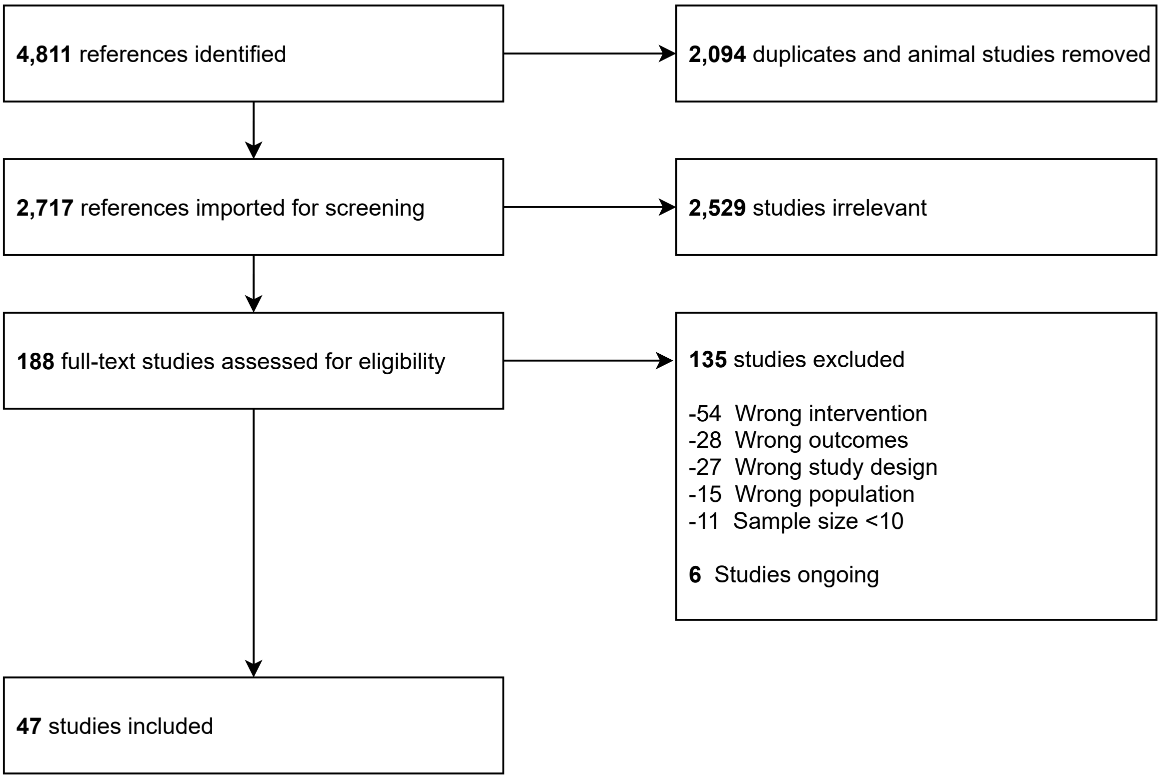

Figure S2: Shannon index changes

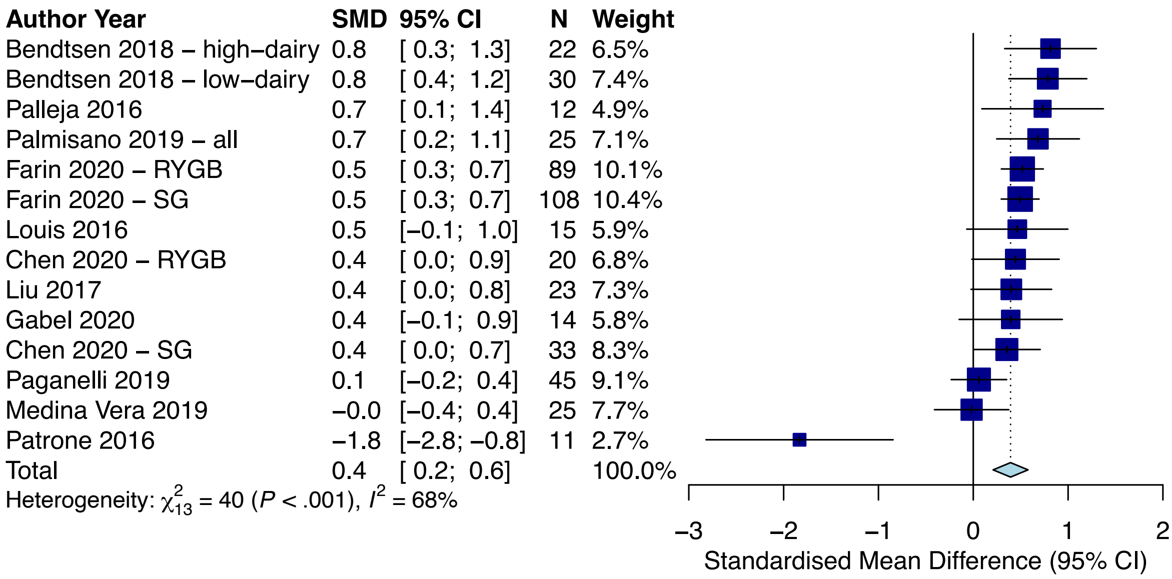

Figure S3: Simpson index: changes

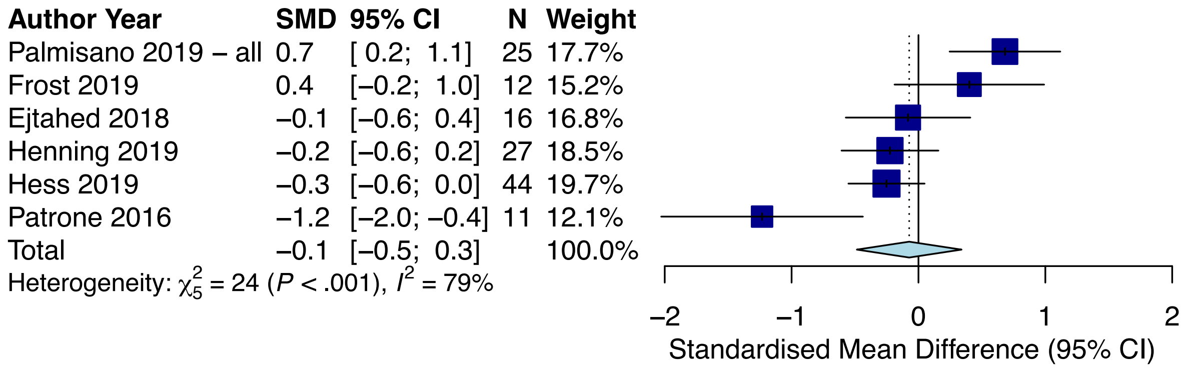

Figure S4: Phylogenetic diversity changes

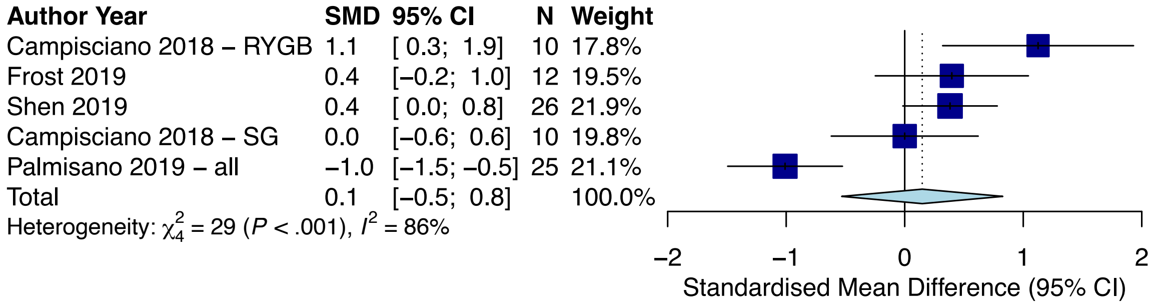

Figure S5: Chao1 changes

Author Year	SMD	95% CI	N	Weight
Campisciano 2018 – GB	1.8	[0.8; 2.8]	10	8.0%
Lin 2019 – SG	1.1	[0.3; 1.9]	10	9.7%
Kong 2013	0.9	[0.4; 1.3]	26	12.3%
Campisciano 2018 – SG	0.1	[−0.5; 0.8]	10	11.0%
Lin 2019 – diet	0.1	[−0.5; 0.8]	10	11.0%
Hess 2019	−0.1	[−0.4; 0.2]	44	13.3%
Henning 2019	−0.3	[−0.6; 0.1]	27	12.7%
Palmisano 2019 – all	−0.5	[−0.9; −0.1]	25	12.5%
Patrone 2016	−1.4	[−2.2; −0.5]	11	9.4%
Total	0.2	[−0.3; 0.6]		100.0%

Heterogeneity: $\chi^2_8 = 52$ ($P < .001$), $I^2 = 84\%$

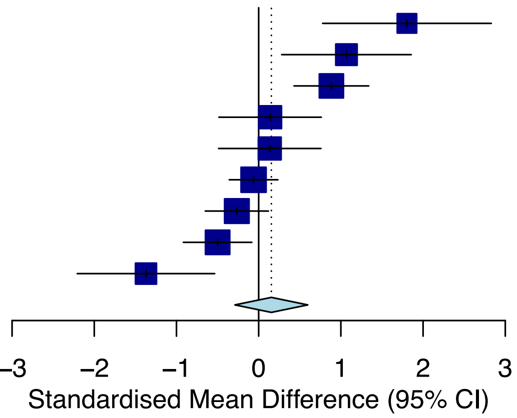

Figure S6: Operational taxonomic units (OTUs) count changes

Author Year	SMD	95% CI	N	Weight
Campisciano 2018 – RYGB	2.0	[0.9; 3.1]	10	6.0%
AlAssal 2020	1.1	[0.4; 1.7]	14	9.8%
Palleja 2016	0.8	[0.2; 1.5]	12	9.8%
Campisciano 2018 – SG	0.5	[−0.2; 1.1]	10	10.0%
Gabel 2020	0.2	[−0.3; 0.7]	14	11.4%
Chen 2020 – all	0.2	[−0.1; 0.5]	53	14.3%
Ejtahed 2018	0.1	[−0.4; 0.6]	16	11.9%
Paganelli 2019	0.0	[−0.3; 0.3]	45	14.1%
Palmisano 2019 – all	−0.6	[−1.0; −0.1]	25	12.7%
Total	0.3	[0.0; 0.7]		100.0%

Heterogeneity: $\chi^2_8 = 35$ ($P < .001$), $I^2 = 77\%$

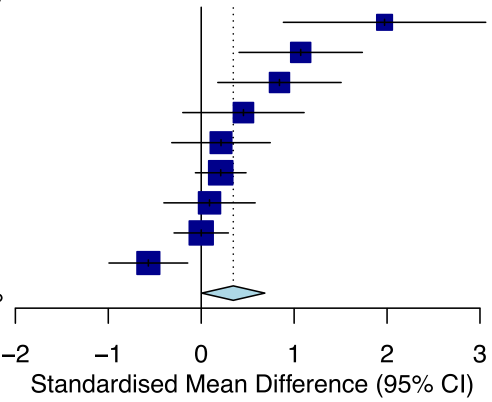

Figure S7: Abundance-based coverage estimator (ACE) index changes

Author Year	SMD	95% CI	N	Weight
Lin 2019 – SG	1.1	[0.3; 1.9]	10	25.6%
Kong 2013	0.5	[−0.1; 1.1]	14	39.4%
Lin 2019 – diet	0.2	[−0.5; 0.8]	10	35.0%
Total	0.5	[0.1; 1.0]		100.0%

Heterogeneity: $\chi^2_2 = 3$ ($P = .18$), $I^2 = 42\%$

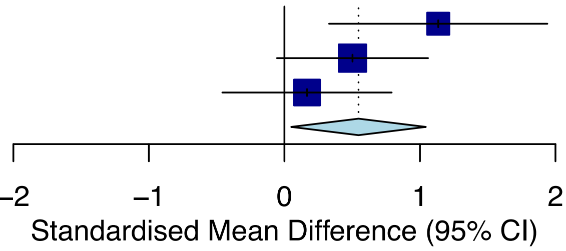

Figure S8: Gene richness changes

Author Year	Baseline	MD	95% CI	N	Weight
Kellerer 2019	5.6 (2.4)	0.1	[−0.4; 0.6]	15	10.2%
vanDielen 2004	134.7 (98.2)	−0.3	[−0.7; 0.1]	27	11.8%
Yang 2014 – RYGB	48.8 (13.8)	−0.4	[−0.7; −0.1]	.	13.0%
Clemente−Postigo 2015 −all	25.2 (8.5)	−0.4	[−0.7; −0.1]	.	13.1%
Nien 2018 – all	30.4 (7)	−0.5	[−0.7; −0.3]	.	14.1%
Yang 2014 – AGB	55.5 (15.8)	−0.8	[−1.5; −0.1]	.	7.7%
Yang 2014 – SG	52 (16.1)	−0.8	[−1.2; −0.4]	.	11.6%
Yang 2014 – MGB	49.1 (16.5)	−1.0	[−1.2; −0.7]	.	13.6%
Mokhtari 2019	36 (11.7)	−2.9	[−4.0; −1.9]	.	4.8%
Total		−0.6	[−0.9; −0.4]		100.0%

Heterogeneity: $\chi^2_8 = 43$ ($P < .001$), $I^2 = 81\%$

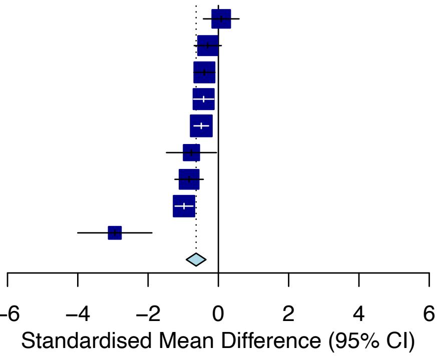

Figure S9: Changes in α-diversity by analytical method used (A) in trials of dietary interventions and (B) in trials of surgical interventions. Tests for between subgroup differences (random effects model): A: dietary trials Q=1.48, p=0.69, B: surgical trials: Q=0.54, p=0.46.

A

Author Year	Follow−up	SMD	95% CI	N	Weight
**Shotgun metagenomic sequencing**					
Louis 2016	2 years	0.5	[−0.1; 1.0]	15	7.4%
Cotillard 2013 − all	3 months	0.2	[−0.1; 0.5]	45	10.8%
Total		0.2	[0.0; 0.5]		18.2%
Heterogeneity: $\chi^2_1 = 1$ (P = .37), $I^2 = 0\%$					
**Culturomics**					
Janczy 2020	3 months	0.2	[−0.3; 0.8]	12	6.9%
Total		0.2	[−0.3; 0.8]		6.9%
Heterogeneity: not applicable					
**16S rRNA gene sequencing − Operaional taxonomic units (OTUs)**					
Bendtsen 2018 − high−dairy	6 months	0.8	[0.3; 1.3]	22	8.1%
Bendtsen 2018 − low−dairy	6 months	0.8	[0.4; 1.2]	30	9.1%
Frost 2019	4 months	0.4	[−0.2; 1.0]	12	6.7%
Gabel 2020	3 months	0.4	[−0.1; 0.9]	14	7.3%
Lin 2019 − diet	3 months	0.2	[−0.5; 0.8]	10	6.3%
Medina Vera 2019	3 months	−0.0	[−0.4; 0.4]	25	9.3%
Henning 2019	3 months	−0.2	[−0.6; 0.2]	27	9.5%
Hess 2019	3 months	−0.3	[−0.6; 0.0]	44	10.7%
Total		0.2	[−0.1; 0.6]		67.0%
Heterogeneity: $\chi^2_7 = 29$ ($P < .001$), $I^2 = 76\%$					
**16S rRNA gene sequencing − Amplicon sequence variant**					
Ejtahed 2018	2 months	−0.1	[−0.6; 0.4]	16	8.0%
Total		−0.1	[−0.6; 0.4]		8.0%
Heterogeneity: not applicable					
Total		0.2	[0.0; 0.4]		100.0%
Heterogeneity: $\chi^2_{11} = 32$ ($P < .001$), $I^2 = 65\%$					

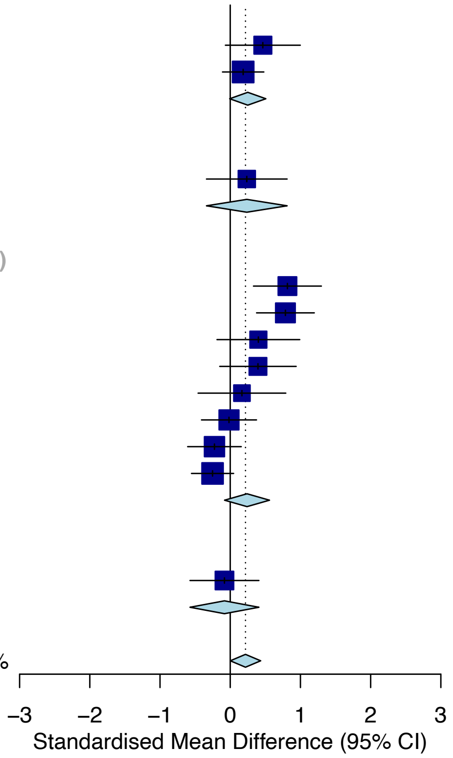

B

Author Year	Follow−up	SMD	95% CI	N	Weight
**Shotgun metagenomic sequencing**					
Aron−Wisnewsky 2019 − AGB	1 year	1.1	[0.3; 1.9]	10	3.5%
Murphy 2017	1 year	0.8	[0.2; 1.4]	14	4.7%
Aron−Wisnewsky 2019 − RYGB	1 year	0.8	[0.2; 1.4]	14	4.8%
Palleja 2016	3 months	0.7	[0.1; 1.4]	12	4.5%
Farin 2020 − RYGB	6 months	0.5	[0.3; 0.8]	89	8.6%
Farin 2020 − SG	6 months	0.5	[0.3; 0.7]	108	8.9%
Liu 2017	3 months	0.4	[0.0; 0.8]	23	6.4%
Total		0.6	[0.4; 0.7]		41.3%
Heterogeneity: $\chi^2_6 = 4$ (P = .66), $I^2 = 0\%$					
**16S rRNA gene sequencing − Operaional taxonomic units (OTUs)**					
Lin 2019 − SG	3 months	1.1	[0.3; 1.9]	10	3.4%
Campisciano 2018 − RYGB	3 months	1.1	[0.3; 1.9]	10	3.4%
Al Assal 2020	1 year	1.1	[0.4; 1.7]	14	4.3%
Kong 2013	6 months	0.9	[0.4; 1.3]	26	6.1%
Palmisano 2019 − all	6 months	0.7	[0.2; 1.1]	25	6.3%
Chen 2020 − RYGB	10 months	0.4	[0.0; 0.9]	20	6.1%
Shen 2019	1 year	0.4	[0.0; 0.8]	26	6.7%
Chen 2020 − SG	10 months	0.4	[0.0; 0.7]	33	7.2%
Paganelli 2019	7 months	0.1	[−0.2; 0.4]	45	7.9%
Campisciano 2018 − SG	3 months	0.0	[−0.6; 0.6]	10	4.6%
Patrone 2016	6 months	−1.8	[−2.8; −0.8]	11	2.5%
Total		0.4	[0.1; 0.7]		58.7%
Heterogeneity: $\chi^2_{10} = 43$ ($P < .001$), $I^2 = 77\%$					
Total		0.5	[0.3; 0.7]		100.0%
Heterogeneity: $\chi^2_{17} = 50$ ($P < .001$), $I^2 = 66\%$					

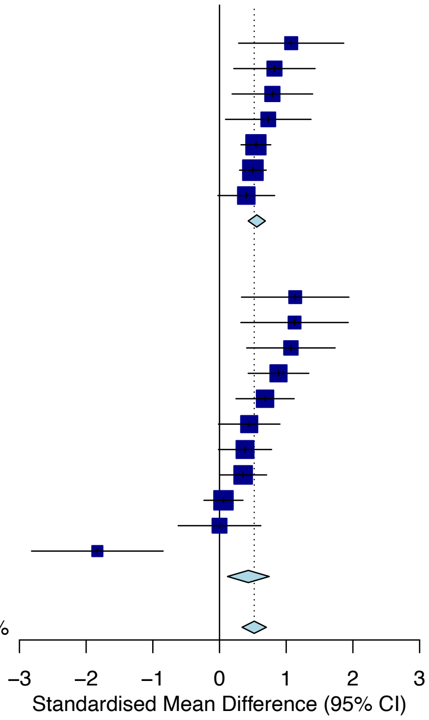

Figure S10: Changes at the taxonomic level of Phylum associated with weight loss

Author Year	Intervention	Baseline	SMD	95% CI	N	Weight
**Actinobacteria**						
Blasco 2017	Diet	6.5 (6.23)	0.2	[−0.4; 0.7]	13	9.9%
Gabel 2020	Diet	6.79 (5.45)	0.1	[−0.4; 0.6]	14	10.4%
Henning 2019	Diet	7.24 (6.07)	−0.0	[−0.4; 0.4]	27	15.5%
Louis 2016	Diet	1.99 (0.88)	0.5	[0.0; 1.1]	14	9.6%
Chen 2020 – SG	Surgery	0.02 (0.27)	0.2	[−0.2; 0.5]	33	17.0%
Chen 2020 – RYGB	Surgery	2.26 (15.14)	−0.1	[−0.5; 0.4]	20	13.1%
Patrone 2016	Surgery	22.5 (17.7)	−0.0	[−0.6; 0.6]	11	8.8%
Sanchez−Alcoholado 2019 – RYGB	Surgery	7.51 (6.55)	−1.0	[−1.7; −0.4]	14	7.7%
Sanchez−Alcoholado 2019 – SG	Surgery	5.8 (5.15)	−0.9	[−1.5; −0.3]	14	8.0%
Total			−0.1	[−0.4; 0.2]		100.0%
Heterogeneity: $\chi^2_8 = 22$ (P = .005), $I^2 = 64\%$						
**Bacteroidetes**						
Blasco 2017	Diet	20.07 (8.73)	0.2	[−0.3; 0.8]	13	7.8%
Gabel 2020	Diet	26.9 (12.2)	−0.1	[−0.6; 0.4]	14	8.3%
Henning 2019	Diet	26.94 (9.83)	0.5	[0.1; 0.9]	27	11.5%
Louis 2016	Diet	67.16 (14.05)	−0.4	[−0.7; 0.0]	14	12.4%
Chen 2020 – SG	Surgery	46.74 (67.5)	−0.1	[−0.4; 0.3]	33	13.7%
Chen 2020 – RYGB	Surgery	48.84 (72.54)	−0.1	[−0.6; 0.3]	20	10.5%
Kikuchi 2018 – SG	Surgery	43.87 (11.61)	0.8	[0.4; 1.3]	22	9.2%
Kikuchi 2018 – SG + DJ bypass	Surgery	45.64 (20.51)	−0.2	[−0.6; 0.3]	18	9.8%
Sanchez−Alcoholado 2019 – RYGB	Surgery	44.43 (15.75)	−0.0	[−0.5; 0.5]	14	8.4%
Sanchez−Alcoholado 2019 – SG	Surgery	41.44 (17.42)	−0.0	[−0.5; 0.5]	14	8.4%
Total			0.1	[−0.2; 0.3]		100.0%
Heterogeneity: $\chi^2_9 = 23$ (P = .007), $I^2 = 61\%$						
**Bacteroidetes/Firmicutes ratio**						
Kikuchi 2018 – SG	Surgery	1.6 (1.05)	0.6	[0.1; 1.0]	22	52.5%
Kikuchi 2018 – SG + DJ bypass	Surgery	1.77 (1.12)	−0.5	[−1.0; 0.0]	18	47.5%
Total			0.0	[−1.0; 1.1]		100.0%
Heterogeneity: $\chi^2_1 = 9$ (P = .002), $I^2 = 89\%$						
**Cyanobacteria**						
Blasco 2017	Diet	0.02 (0.01)	−0.3	[−0.9; 0.2]	13	24.1%
Chen 2020 – SG	Surgery	0.01 (0.35)	0.0	[−0.3; 0.4]	33	43.0%
Chen 2020 – RYGB	Surgery	0.05 (1.39)	0.1	[−0.4; 0.5]	20	32.9%
Total			−0.0	[−0.3; 0.2]		100.0%
Heterogeneity: $\chi^2_2 = 1$ (P = .48), $I^2 = 0\%$						
**Elusimicrobia**						
Blasco 2017	Diet	0 (0)	0.0	[−0.5; 0.5]	13	24.8%
Chen 2020 – SG	Surgery	0 (0)	0.0	[−0.3; 0.4]	33	42.6%
Chen 2020 – RYGB	Surgery	0 (0)	0.1	[−0.4; 0.5]	20	32.6%
Total			0.0	[−0.2; 0.3]		100.0%
Heterogeneity: $\chi^2_2 = 0$ (P = .98), $I^2 = 0\%$						
**Epsilonbacteraeota**						
Chen 2020 – SG	Surgery	0 (0)	0.0	[−0.3; 0.4]	33	56.6%
Chen 2020 – RYGB	Surgery	0 (0)	0.1	[−0.4; 0.5]	20	43.4%
Total			0.0	[−0.2; 0.3]		100.0%
Heterogeneity: $\chi^2_1 = 0$ (P = .95), $I^2 = 0\%$						
**Euryarchaeota**						
Gabel 2020	Diet	0.44 (4.45)	0.0	[−0.5; 0.5]	14	40.5%
Henning 2019	Diet	1.76 (2.95)	−0.2	[−0.5; 0.2]	27	59.5%
Total			−0.1	[−0.4; 0.2]		100.0%
Heterogeneity: $\chi^2_1 = 0$ (P = .57), $I^2 = 0\%$						
**Firmicutes**						
Blasco 2017	Diet	66.34 (14.17)	−0.1	[−0.7; 0.4]	13	8.5%
Gabel 2020	Diet	61.2 (12.2)	0.1	[−0.4; 0.6]	14	9.0%
Henning 2019	Diet	61.29 (11)	−0.4	[−0.8; 0.0]	27	12.8%
Louis 2016	Diet	28.56 (13.93)	0.1	[−0.4; 0.6]	14	9.0%
Chen 2020 – SG	Surgery	39.68 (72.14)	0.0	[−0.3; 0.4]	33	14.7%
Chen 2020 – RYGB	Surgery	36.86 (71.54)	−0.0	[−0.4; 0.4]	20	11.3%
Kikuchi 2018 – SG	Surgery	33.69 (10.93)	−0.5	[−0.9; 0.0]	22	11.2%
Patrone 2016	Surgery	67.58 (15.82)	0.5	[−0.1; 1.1]	11	7.0%
Sanchez−Alcoholado 2019 – RYGB	Surgery	31.7 (16.65)	−0.4	[−1.0; 0.1]	14	8.5%
Sanchez−Alcoholado 2019 – SG	Surgery	35.84 (14.28)	−0.6	[−1.2; 0.0]	14	7.9%
Total			−0.1	[−0.3; 0.0]		100.0%
Heterogeneity: $\chi^2_9 = 14$ (P = .11), $I^2 = 38\%$						
**Firmicutes/Bacteroidetes ratio**						
Remely 2015	Diet	2.32 (1.44)	−0.7	[−1.2; −0.2]	20	52.1%
AlAssal 2020	Surgery	1.58 (3.29)	−0.2	[−0.7; 0.3]	14	47.9%
Total			−0.5	[−1.0; 0.1]		100.0%
Heterogeneity: $\chi^2_1 = 2$ (P = .14), $I^2 = 55\%$						
**Fusobacteria**						
Blasco 2017	Diet	0.11 (0.02)	−0.2	[−0.8; 0.3]	13	19.4%
Gabel 2020	Diet	0.06 (0.26)	−0.2	[−0.7; 0.3]	14	20.5%
Chen 2020 – SG	Surgery	2.55 (35.14)	−0.0	[−0.3; 0.3]	33	34.1%
Chen 2020 – RYGB	Surgery	2.18 (28.63)	0.1	[−0.3; 0.5]	20	26.0%
Total			−0.1	[−0.3; 0.2]		100.0%
Heterogeneity: $\chi^2_3 = 1$ (P = .72), $I^2 = 0\%$						
**Kiritimatiellaeota**						
Chen 2020 – SG	Surgery	0 (0)	−0.0	[−0.6; 0.5]	33	43.0%
Chen 2020 – RYGB	Surgery	0 (0)	0.1	[−0.4; 0.5]	20	57.0%
Total			0.0	[−0.3; 0.4]		100.0%
Heterogeneity: $\chi^2_1 = 0$ (P = .85), $I^2 = 0\%$						
**Lentisphaerae**						
Chen 2020 – SG	Surgery	0.01 (0.22)	0.1	[−0.3; 0.4]	33	56.6%
Chen 2020 – RYGB	Surgery	0 (0.06)	0.1	[−0.4; 0.5]	20	43.4%
Total			0.1	[−0.2; 0.3]		100.0%
Heterogeneity: $\chi^2_1 = 0$ (P = .95), $I^2 = 0\%$						
**Patescibacteria**						
Chen 2020 – SG	Surgery	0 (0.03)	0.1	[−0.2; 0.4]	33	56.6%
Chen 2020 – RYGB	Surgery	0 (0.04)	0.1	[−0.4; 0.5]	20	43.4%
Total			0.1	[−0.2; 0.4]		100.0%
Heterogeneity: $\chi^2_1 = 0$ (P = .88), $I^2 = 0\%$						
**Proteobacteria**						
Blasco 2017	Diet	2.94 (1.08)	0.0	[−0.5; 0.6]	13	11.2%
Gabel 2020	Diet	2.13 (1.01)	−0.1	[−0.6; 0.5]	14	11.8%
Henning 2019	Diet	1.09 (1.61)	−0.0	[−0.4; 0.4]	27	17.5%
Louis 2016	Diet	0.4 (0.29)	1.5	[0.7; 2.2]	14	6.7%
Chen 2020 – SG	Surgery	8.51 (47.36)	0.0	[−0.3; 0.4]	33	19.3%
Chen 2020 – RYGB	Surgery	9.79 (33.44)	0.1	[−0.4; 0.5]	20	14.8%
Sanchez−Alcoholado 2019 – RYGB	Surgery	15.46 (12.68)	0.9	[0.3; 1.6]	14	9.0%
Sanchez−Alcoholado 2019 – SG	Surgery	16.32 (14.22)	0.8	[0.2; 1.4]	14	9.8%
Campisciano 2018 – RYGB	.	.			10	0.0%
Total			0.3	[0.0; 0.7]		100.0%
Heterogeneity: $\chi^2_7 = 23$ (P = .002), $I^2 = 70\%$						
**Synergistetes**						
Blasco 2017	Diet	0.11 (0.06)	0.1	[−0.5; 0.6]	13	24.7%
Chen 2020 – SG	Surgery	0 (0.08)	0.0	[−0.3; 0.4]	33	42.6%
Chen 2020 – RYGB	Surgery	0.01 (0.11)	−0.0	[−0.5; 0.4]	20	32.7%
Total			0.0	[−0.2; 0.3]		100.0%
Heterogeneity: $\chi^2_2 = 0$ (P = .97), $I^2 = 0\%$						
**Tenericutes**						
Blasco 2017	Diet	0.03 (0.01)	−0.2	[−0.7; 0.4]	13	19.6%
Gabel 2020	Diet	0.09 (0.3)	0.2	[−0.3; 0.8]	14	20.4%
Chen 2020 – SG	Surgery	0.02 (0.69)	0.0	[−0.3; 0.4]	33	34.0%
Chen 2020 – RYGB	Surgery	0.01 (0.21)	0.1	[−0.4; 0.5]	20	26.1%
Total			0.1	[−0.2; 0.3]		100.0%
Heterogeneity: $\chi^2_3 = 1$ (P = .75), $I^2 = 0\%$						
**Verrucomicrobia**						
Blasco 2017	Diet	0.02 (0.07)	0.2	[−0.3; 0.8]	13	10.2%
Gabel 2020	Diet	2.33 (4.39)	−0.0	[−0.6; 0.5]	14	10.9%
Henning 2019	Diet	0.75 (1.9)	−0.1	[−0.5; 0.3]	27	16.2%
Louis 2016	Diet	.	0.3	[−0.2; 0.9]	14	10.5%
Chen 2020 – SG	Surgery	0.01 (0.21)	0.1	[−0.3; 0.4]	33	17.7%
Chen 2020 – RYGB	Surgery	0.01 (0.11)	0.1	[−0.4; 0.5]	20	13.7%
Sanchez−Alcoholado 2019 – RYGB	Surgery	0.03 (0.11)	0.5	[−0.1; 1.0]	14	10.1%
Sanchez−Alcoholado 2019 – SG	Surgery	0.14 (0.37)	0.3	[−0.3; 0.8]	14	10.7%
Total			0.1	[0.0; 0.3]		100.0%
Heterogeneity: $\chi^2_7 = 5$ (P = .67), $I^2 = 0\%$						

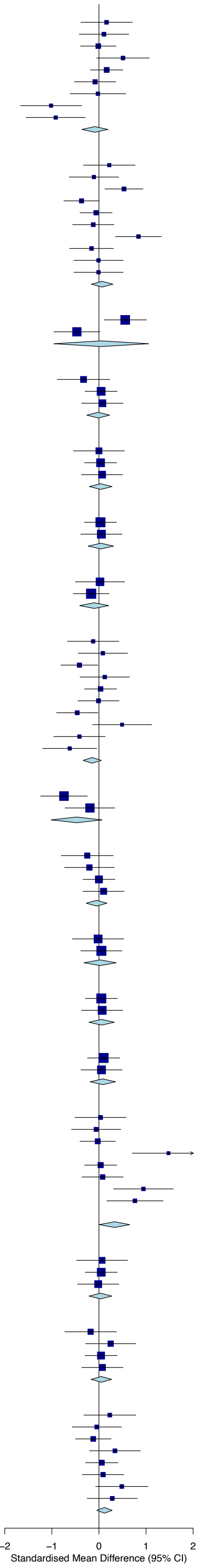

Figure S11: Changes in Actinobacteria by sequencing method. Test for between subgroup differences (random effects model): Q=20.29, p<0.0001

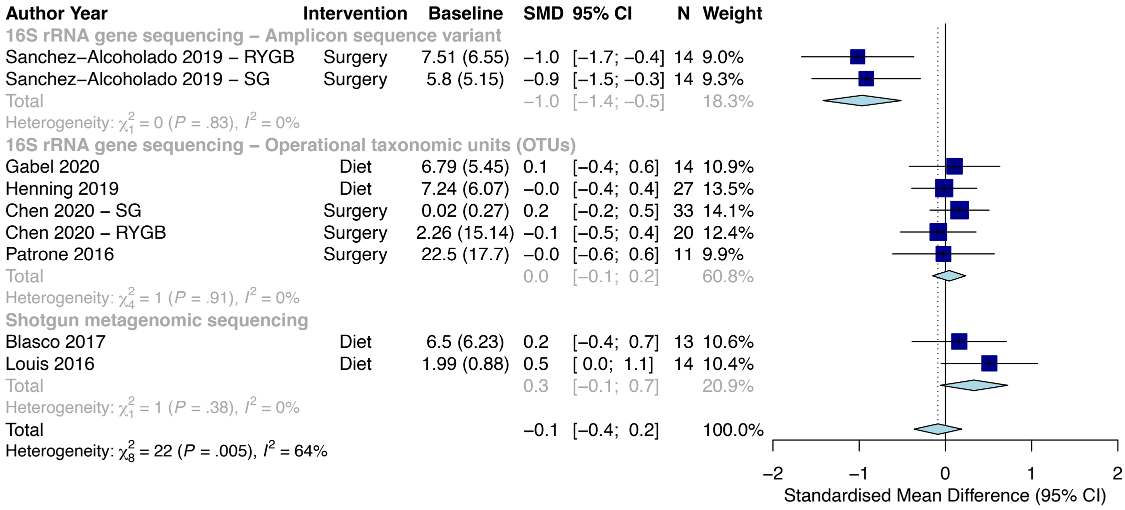

Figure S12: Changes in Bacteroidetes by sequencing method. Test for between subgroup differences (random effects model): Q=0.78, p=0.68

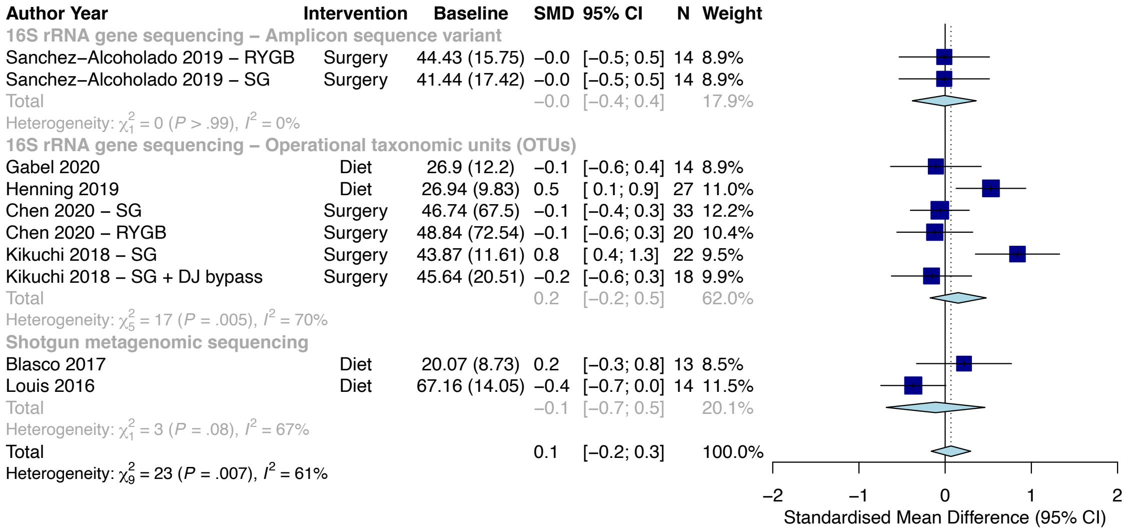

Figure S13: Changes in Firmicutes by sequencing method. Test for between subgroup differences (random effects model): Q=4.10, p=0.13

Author Year	Intervention	Baseline	SMD	95% CI	N	Weight
**16S rRNA gene sequencing – Amplicon sequence variant**						
Sanchez–Alcoholado 2019 – RYGB	Surgery	31.7 (16.65)	–0.4	[–1.0; 0.1]	14	8.5%
Sanchez–Alcoholado 2019 – SG	Surgery	35.84 (14.28)	–0.6	[–1.2; 0.0]	14	8.0%
Total			–0.5	[–0.9; –0.1]		16.5%
Heterogeneity: $\chi^2_1 = 0$ (P = .61), $I^2 = 0\%$						
**16S rRNA gene sequencing – Operational taxonomic units (OTUs)**						
Gabel 2020	Diet	61.2 (12.2)	0.1	[–0.4; 0.6]	14	9.0%
Henning 2019	Diet	61.29 (11)	–0.4	[–0.8; 0.0]	27	12.7%
Chen 2020 – SG	Surgery	39.68 (72.14)	0.0	[–0.3; 0.4]	33	14.7%
Chen 2020 – RYGB	Surgery	36.86 (71.54)	–0.0	[–0.4; 0.4]	20	11.3%
Kikuchi 2018 – SG	Surgery	33.69 (10.93)	–0.5	[–0.9; 0.0]	22	11.2%
Patrone 2016	Surgery	67.58 (15.82)	0.5	[–0.1; 1.1]	11	7.0%
Total			–0.1	[–0.3; 0.2]		66.0%
Heterogeneity: $\chi^2_5 = 10$ (P = .08), $I^2 = 48\%$						
**Shotgun metagenomic sequencing**						
Blasco 2017	Diet	66.34 (14.17)	–0.1	[–0.7; 0.4]	13	8.6%
Louis 2016	Diet	28.56 (13.93)	0.1	[–0.4; 0.6]	14	9.0%
Total			0.0	[–0.4; 0.4]		17.6%
Heterogeneity: $\chi^2_1 = 0$ (P = .52), $I^2 = 0\%$						
Total			–0.1	[–0.3; 0.0]		100.0%
Heterogeneity: $\chi^2_9 = 14$ (P = .11), $I^2 = 38\%$						

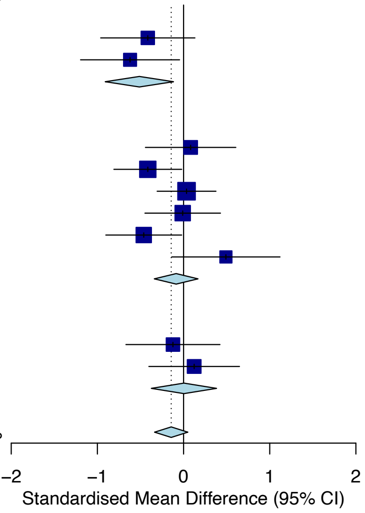

Figure S14: Changes in Proteobacteria by sequencing method. Test for between subgroup differences (random effects model): Q=12.25, p=0.002

Author Year	Intervention	Baseline	SMD	95% CI	N	Weight
**16S rRNA gene sequencing – Amplicon sequence variant**						
Sanchez–Alcoholado 2019 – RYGB	Surgery	15.46 (12.68)	0.9	[0.3; 1.6]	14	10.8%
Sanchez–Alcoholado 2019 – SG	Surgery	16.32 (14.22)	0.8	[0.2; 1.4]	14	11.3%
Total			0.8	[0.4; 1.3]		22.1%
Heterogeneity: $\chi^2_1 = 0$ (P = .68), $I^2 = 0\%$						
**16S rRNA gene sequencing – Operational taxonomic units (OTUs)**						
Gabel 2020	Diet	2.13 (1.01)	–0.1	[–0.6; 0.5]	14	12.5%
Henning 2019	Diet	1.09 (1.61)	–0.0	[–0.4; 0.4]	27	14.9%
Chen 2020 – SG	Surgery	8.51 (47.36)	0.0	[–0.3; 0.4]	33	15.4%
Chen 2020 – RYGB	Surgery	9.79 (33.44)	0.1	[–0.4; 0.5]	20	13.9%
Total			0.0	[–0.2; 0.2]		56.6%
Heterogeneity: $\chi^2_3 = 0$ (P = .97), $I^2 = 0\%$						
**Shotgun metagenomic sequencing**						
Blasco 2017	Diet	2.94 (1.08)	0.0	[–0.5; 0.6]	13	12.2%
Louis 2016	Diet	0.4 (0.29)	1.5	[0.7; 2.2]	14	9.0%
Total			0.7	[–0.7; 2.1]		21.2%
Heterogeneity: $\chi^2_1 = 9$ (P = .003), $I^2 = 89\%$						
Total			0.3	[0.0; 0.7]		100.0%
Heterogeneity: $\chi^2_7 = 23$ (P = .002), $I^2 = 70\%$						

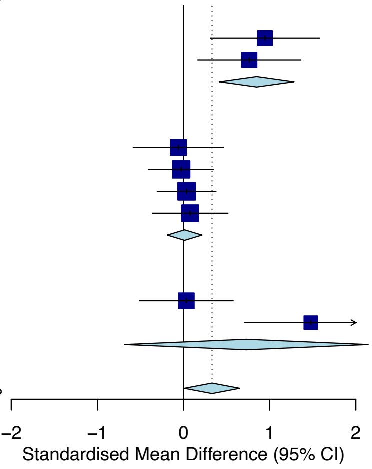

Figure S15: Changes in Verrucomicrobia by sequencing method. Test for between subgroup differences (random effects model): Q=3.86, p=0.15

Author Year	Intervention	Baseline	SMD	95% CI	N	Weight
**16S rRNA gene sequencing – Amplicon sequence variant**						
Sanchez–Alcoholado 2019 – RYGB	Surgery	0.03 (0.11)	0.5	[−0.1; 1.0]	14	8.6%
Sanchez–Alcoholado 2019 – SG	Surgery	0.14 (0.37)	0.3	[−0.3; 0.8]	14	9.3%
Total			0.4	[0.0; 0.8]		17.9%
Heterogeneity: $\chi^2_1 = 0$ (P = .61), $I^2 = 0\%$						
**16S rRNA gene sequencing – Operational taxonomic units (OTUs)**						
Gabel 2020	Diet	2.33 (4.39)	−0.0	[−0.6; 0.5]	14	9.7%
Henning 2019	Diet	0.75 (1.9)	−0.1	[−0.5; 0.3]	27	18.5%
Chen 2020 – SG	Surgery	0.01 (0.21)	0.1	[−0.3; 0.4]	33	22.2%
Chen 2020 – RYGB	Surgery	0.01 (0.11)	0.1	[−0.4; 0.5]	20	13.8%
Total			−0.0	[−0.2; 0.2]		64.2%
Heterogeneity: $\chi^2_3 = 1$ (P = .88), $I^2 = 0\%$						
**Shotgun metagenomic sequencing**						
Blasco 2017	Diet	0.02 (0.07)	0.2	[−0.3; 0.8]	13	8.7%
Louis 2016	Diet	.	0.3	[−0.2; 0.9]	14	9.1%
Total			0.3	[−0.1; 0.7]		17.9%
Heterogeneity: $\chi^2_1 = 0$ (P = .78), $I^2 = 0\%$						
Total			0.1	[0.0; 0.3]		100.0%
Heterogeneity: $\chi^2_7 = 5$ (P = .67), $I^2 = 0\%$						

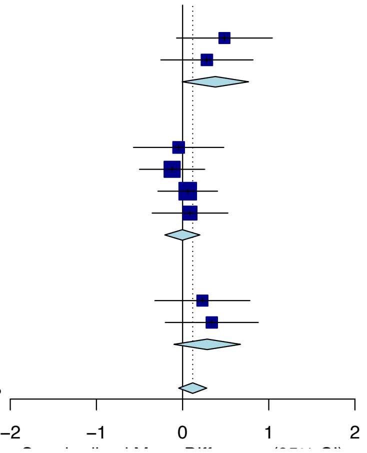

Figure S16: Changes at the taxonomic level of Genus associated with weight loss

Author Year	Intervention	SMD	95% CI	N	Weight
**Akkermansia**					
Chen 2020 – RYGB	Surgery	0.5	[0.0; 0.9]	20	25.0%
Chen 2020 – SG	Surgery	0.4	[0.1; 0.8]	33	26.1%
Sanchez–Alcoholado 2019 – SG	Surgery	0.6	[0.0; 1.1]	14	23.7%
Shen 2019	Surgery	0.6	[0.2; 1.1]	23	25.2%
Total		0.5	[0.3; 0.7]		100.0%
Heterogeneity: $\chi^2_3 = 0$ ($P = .94$), $I^2 = 0\%$					
**Bacteroides**					
Frost 2019	Diet	0.1	[–0.5; 0.7]	12	19.7%
Henning 2019	Diet	0.4	[0.0; 0.8]	27	21.4%
Janczy 2020	Diet	0.0	[–0.5; 0.6]	12	19.7%
Simoes 2014	Diet	0.4	[–0.2; 0.9]	16	19.9%
Kong 2013	Surgery	0.8	[0.2; 1.4]	14	19.3%
Total		0.3	[0.1; 0.6]		100.0%
Heterogeneity: $\chi^2_4 = 4$ ($P = .44$), $I^2 = 0\%$					
**Bifidobacterium**					
Binkworth 2020 high–fat low–carb	Diet	–1.7	[–1.9; –1.4]	48	11.0%
Binkworth 2020 low–fat high–carb	Diet	0.2	[–0.1; 0.5]	43	10.7%
Hess 2019	Diet	–0.1	[–0.4; 0.2]	44	10.8%
Janczy 2020	Diet	–0.4	[–1.0; 0.2]	12	9.4%
Simoes 2014	Diet	–0.3	[–0.9; 0.2]	16	9.7%
Chen 2017	Surgery			24	0.0%
Kikuchi 2020 SG	Surgery	–0.2	[–0.7; 0.2]	22	10.3%
Kikuchi 2020 SG +DB	Surgery	–0.9	[–1.5; –0.4]	18	9.6%
Kong 2013	Surgery	–0.4	[–1.0; 0.1]	14	9.6%
Sanchez–Alcoholado 2019 – RYGB	Surgery	–0.8	[–1.4; –0.2]	14	9.4%
Sanchez–Alcoholado 2019 – SG	Surgery	–0.6	[–1.2; –0.1]	14	9.5%
Total		–0.5	[–1.0; 0.0]		100.0%
Heterogeneity: $\chi^2_9 = 132$ ($P < .001$), $I^2 = 93\%$					
**Butyricimonas**					
Frost 2019	Diet	–0.3	[–0.8; 0.3]	12	24.6%
Aron–Wisnewsky 2019 – AGB	Surgery	0.4	[–0.2; 1.0]	10	23.6%
Aron–Wisnewsky 2019 – RYGB	Surgery	0.5	[0.0; 1.1]	14	24.8%
Chen 2020 – SG	Surgery	0.8	[0.4; 1.2]	33	26.9%
Total		0.4	[–0.1; 0.9]		100.0%
Heterogeneity: $\chi^2_3 = 9$ ($P = .03$), $I^2 = 67\%$					
**Granulicatella**					
Frost 2019	Diet	–0.5	[–1.1; 0.1]	12	23.7%
Chen 2020 – RYGB	Surgery	0.6	[0.1; 1.1]	20	25.4%
Chen 2020 – SG	Surgery	0.5	[0.2; 0.9]	33	26.7%
Sanchez–Alcoholado 2019 – RYGB	Surgery	0.6	[0.0; 1.1]	14	24.2%
Total		0.3	[–0.1; 0.8]		100.0%
Heterogeneity: $\chi^2_3 = 10$ ($P = .02$), $I^2 = 71\%$					
**Lactobacillus**					
Binkworth 2020 high–fat low–carb	Diet	–0.7	[–1.0; –0.3]	48	15.1%
Binkworth 2020 low–fat high–carb	Diet	–0.1	[–0.4; 0.2]	43	15.1%
Janczy 2020	Diet	–0.4	[–1.0; 0.2]	12	13.3%
Remely 2020	Diet	0.7	[0.2; 1.2]	20	14.0%
Simoes 2014	Diet	–0.6	[–1.1; 0.0]	16	13.4%
Chen 2017	Surgery	–0.1	[–0.6; 0.3]	24	14.6%
Chen 2017	Surgery	–0.1	[–0.6; 0.3]	24	14.6%
Kong 2013	Surgery			26	0.0%
Total		–0.2	[–0.5; 0.1]		100.0%
Heterogeneity: $\chi^2_6 = 23$ ($P < .001$), $I^2 = 74\%$					

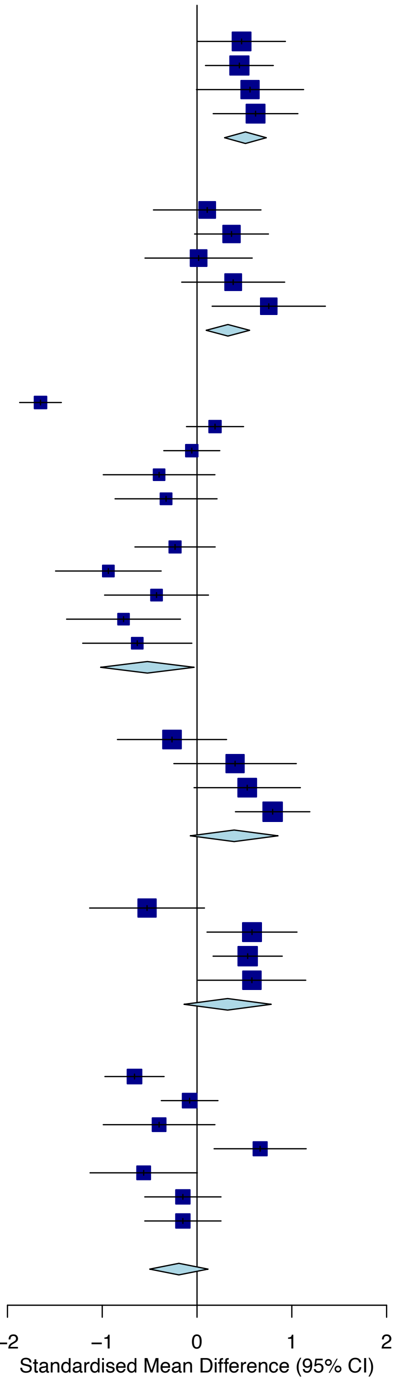

Figure S17: Changes in *Bifidobacterium* by analytical method. Test for between subgroup differences (random effects model): Q=1.86, p=0.60

Author Year	Intervention	SMD	95% CI	N	Weight
**16S rRNA gene sequencing – Amplicon sequence variant**					
Sanchez–Alcoholado 2019 – RYGB	Surgery	−0.8	[−1.4; −0.2]	14	9.5%
Sanchez–Alcoholado 2019 – SG	Surgery	−0.6	[−1.2; −0.1]	14	9.6%
Total		−0.7	[−1.1; −0.3]		19.1%
Heterogeneity: $\chi^2_1 = 0$ (P = .73), $I^2 = 0\%$					
**16S rRNA gene sequencing – Operational taxonomic units (OTUs)**					
Hess 2019	Diet	−0.1	[−0.4; 0.2]	44	10.6%
Chen 2017	Surgery			24	0.0%
Kikuchi 2020 SG	Surgery	−0.2	[−0.7; 0.2]	22	10.2%
Kikuchi 2020 SG +DB	Surgery	−0.9	[−1.5; −0.4]	18	9.7%
Kong 2013	Surgery	−0.4	[−1.0; 0.1]	14	9.7%
Total		−0.4	[−0.7; 0.0]		40.2%
Heterogeneity: $\chi^2_3 = 8$ (P = .05), $I^2 = 62\%$					
**Culturomics**					
Binkworth 2020 high–fat low–carb	Diet	−1.7	[−1.9; −1.4]	48	10.8%
Binkworth 2020 low–fat high–carb	Diet	0.2	[−0.1; 0.5]	43	10.6%
Janczy 2020	Diet	−0.4	[−1.0; 0.2]	12	9.5%
Total		−0.6	[−2.0; 0.7]		30.9%
Heterogeneity: $\chi^2_2 = 97$ ($P < .001$), $I^2 = 98\%$					
**Denaturing gradient gel electrophoresis**					
Simoes 2014	Diet	−0.3	[−0.9; 0.2]	16	9.8%
Total		−0.3	[−0.9; 0.2]		9.8%
Heterogeneity: not applicable					
Total		−0.5	[−1.0; 0.0]		100.0%
Heterogeneity: $\chi^2_9 = 132$ ($P < .001$), $I^2 = 93\%$					

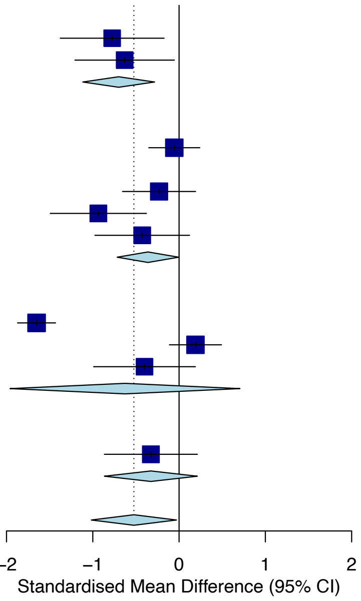

Figure S18: Changes in *Lactobacillus* by analytical method. Test for between subgroup differences (random effects model): Q=3.47, p=0.18

Author Year	Intervention	SMD	95% CI	N	Weight
**16S rRNA gene sequencing – Operational taxonomic units (OTUs)**					
Remely 2020	Diet	0.7	[0.2; 1.2]	20	13.3%
Chen 2017	Surgery	−0.1	[−0.6; 0.3]	24	14.9%
Chen 2017	Surgery	−0.1	[−0.6; 0.3]	24	14.9%
Kong 2013	Surgery			26	0.0%
Total		0.1	[−0.4; 0.6]		43.1%
Heterogeneity: $\chi^2_2 = 8$ (P = .02), $I^2 = 75\%$					
**Culturomics**					
Binkworth 2020 high–fat low–carb	Diet	−0.7	[−1.0; −0.3]	48	16.6%
Binkworth 2020 low–fat high–carb	Diet	−0.1	[−0.4; 0.2]	43	16.8%
Janczy 2020	Diet	−0.4	[−1.0; 0.2]	12	11.5%
Total		−0.4	[−0.8; 0.0]		44.9%
Heterogeneity: $\chi^2_2 = 7$ (P = .03), $I^2 = 71\%$					
**Denaturing gradient gel electrophoresis**					
Simoes 2014	Diet	−0.6	[−1.1; 0.0]	16	11.9%
Total		−0.6	[−1.1; 0.0]		11.9%
Heterogeneity: not applicable					
Total		−0.2	[−0.5; 0.1]		100.0%
Heterogeneity: $\chi^2_6 = 23$ ($P < .001$), $I^2 = 74\%$					

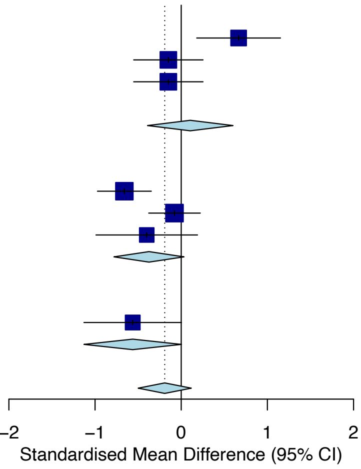

Figure S19: Changes in faecal short-chain fatty acids associated with weight loss

A: Acetate (mmol/l)

Author Year	Baseline	MD	95% CI	N	Time	Weight
Ejtahed 2018	80.8 (27.2)	15.2	[2.1; 28.3]	16	2 months	15.9%
Brinkworth 2009 – high–carb low–fat	62.4 (18.5)	3.3	[–2.8; 9.4]	43	2 months	21.8%
Aasbrenn/Farup 2020	7.2 (4.3)	–3.8	[–6.3; –1.2]	80	1 year	23.7%
Brinkworth 2009 – low–carb high–fat	58.5 (19.1)	–10.6	[–17.3; –3.9]	48	2 months	21.3%
Patrone 2016	70.3 (21.9)	–30.3	[–41.8; –18.8]	11	6 months	17.3%
Total		–5.3	[–14.2; 3.7]			100.0%

Heterogeneity: $\chi^2_4 = 38$ ($P < .001$), $I^2 = 89\%$

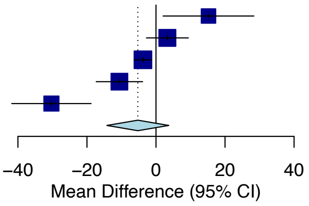

B: Butyrate (mmol/l)

Author Year	Baseline	MD	95% CI	N	Time	Weight
Ejtahed 2018	18.5 (9.5)	7.2	[1.3; 13.2]	16	2 months	11.7%
Patrone 2016	17 (5.8)	2.5	[–4.3; 9.3]	11	6 months	9.7%
Brinkworth 2009 – high–carb low–fat	21.1 (8.9)	–0.5	[–3.4; 2.4]	43	2 months	23.6%
Aasbrenn/Farup 2020	7.2 (4.3)	–1.3	[–2.5; –0.1]	80	1 year	31.7%
Brinkworth 2009 – low–carb high–fat	18.3 (9.7)	–3.9	[–6.8; –1.0]	48	2 months	23.4%
Total		–0.4	[–2.9; 2.1]			100.0%

Heterogeneity: $\chi^2_4 = 13$ ($P = .01$), $I^2 = 68\%$

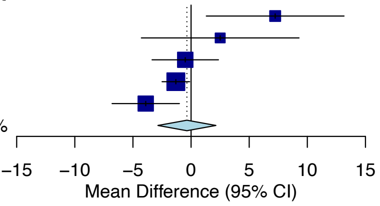

C: Propionate (mmol/l)

Author Year	Baseline	MD	95% CI	N	Time	Weight
Ejtahed 2018	19 (10.9)	4.0	[–0.8; 8.9]	16	2 months	15.2%
Brinkworth 2009 – high–carb low–fat	21.4 (7.6)	–0.8	[–3.2; 1.6]	43	2 months	23.1%
Aasbrenn/Farup 2020	6.5 (3.7)	–1.0	[–2.0; 0.0]	80	1 year	26.9%
Brinkworth 2009 – low–carb high–fat	17.6 (7.6)	–1.9	[–4.2; 0.4]	48	2 months	23.6%
Patrone 2016	26.4 (12.3)	–15.2	[–21.6; –8.8]	11	6 months	11.3%
Total		–2.0	[–4.8; 0.8]			100.0%

Heterogeneity: $\chi^2_4 = 23$ ($P < .001$), $I^2 = 83\%$

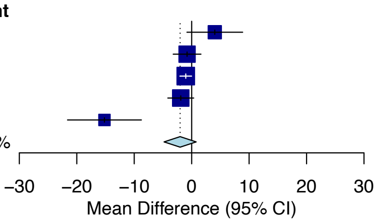

D: Total SCFA (mmol/l)

Author Year	Baseline	MD	95% CI	N	Time	Weight
Brinkworth 2009 – high–carb low–fat	113.1 (31.8)	1.4	[–9.2; 12.0]	43	2 months	25.7%
Aasbrenn/Farup 2020	37 (17.5)	–5.6	[–10.4; –0.8]	80	1 year	48.7%
Brinkworth 2009 – low–carb high–fat	102.2 (33.5)	–15.8	[–27.4; –4.2]	48	2 months	22.8%
Patrone 2016	112.7 (74.5)	–18.8	[–59.6; 22.0]	11	6 months	2.8%
Total		–6.5	[–13.4; 0.4]			100.0%

Heterogeneity: $\chi^2_3 = 5$ ($P = .17$), $I^2 = 40\%$

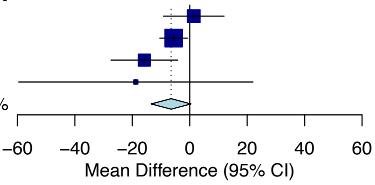

Figure S20: Changes in lipopolysaccharide binding protein associated with weight loss

Author Year	Baseline	MD	95% CI	N	Weight
Kellerer 2019	5.6 (2.4)	0.1	[−0.4; 0.6]	15	10.2%
vanDielen 2004	134.7 (98.2)	−0.3	[−0.7; 0.1]	27	11.8%
Yang 2014 − RYGB	48.8 (13.8)	−0.4	[−0.7; −0.1]	47	13.0%
Clemente−Postigo 2015 −all	25.2 (8.5)	−0.4	[−0.7; −0.1]	50	13.1%
Nien 2018 − all	30.4 (7)	−0.5	[−0.7; −0.3]	101	14.1%
Yang 2014 − AGB	55.5 (15.8)	−0.8	[−1.5; −0.1]	10	7.7%
Yang 2014 − SG	52 (16.1)	−0.8	[−1.2; −0.4]	32	11.6%
Yang 2014 − MGB	49.1 (16.5)	−1.0	[−1.2; −0.7]	89	13.6%
Mokhtari 2019	36 (11.7)	−2.9	[−4.0; −1.9]	23	4.8%
Total		−0.6	[−0.9; −0.4]		100.0%

Heterogeneity: $\chi^2_8 = 43$ ($P < .001$), $I^2 = 81\%$

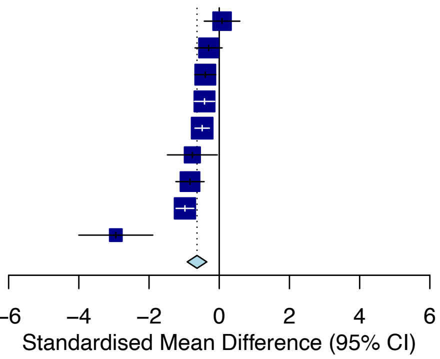

Figure S21: Changes in lipopolysaccharide associated with weight loss

Author Year	Baseline	SMD	95% CI	N	Weight
Clemente−Postigo 2015 − all	14.6 (7.5)	−0.4	[−0.7; −0.1]	50	43.0%
Troseid 2013	83.5 (27.4)	−0.5	[−0.8; −0.2]	49	42.0%
Monte 2012	56.7 (13)	−1.1	[−1.7; −0.4]	15	15.0%
Total		−0.5	[−0.8; −0.3]		100.0%

Heterogeneity: $\chi^2_2 = 3$ ($P = .19$), $I^2 = 41\%$

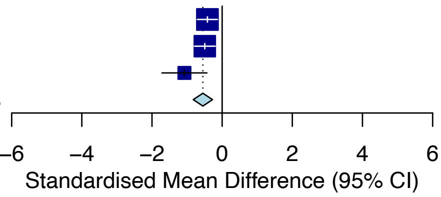

Figure S22: Changes in the lactulose:mannitol ratio associated with weight loss

Author Year	Baseline	SMD	95% CI	N	Weight
Obermayer 2021	0.01 (0.01)	−0.7	[−1.3; 0.0]	10	37.2%
Kellerer 2019	0.03 (0.01)	−0.7	[−1.2; −0.1]	16	38.7%
Damms−Machado 2017	0.1 (0.01)	−4.1	[−5.8; −2.4]	13	24.1%
Total		−1.5	[−2.8; −0.2]		100.0%

Heterogeneity: $\chi^2_2 = 15$ ($P < .001$), $I^2 = 86\%$

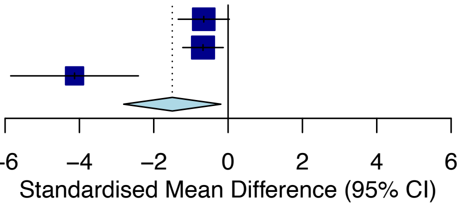

Figure S23: Changes in faecal calprotectin (mcg/g), a marker of faecal inflammation.

Author Year	Baseline	MD	95% CI	N	Weight
Wilbrink 2020	3.7 (5.4)	11.9	[9.2; 14.6]	14	53.5%
Kant 2013 − all	42.1 (73.6)	−26.3	[−46.3; −6.3]	39	46.5%
Total		−5.9	[−43.2; 31.5]		100.0%

Heterogeneity: $\chi^2_1 = 14$ ($P < .001$), $I^2 = 93\%$

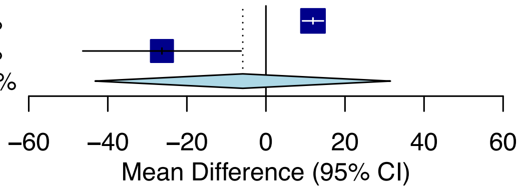

Figure S24: A-diversity changes among studies with lower risk of bias

Author Year	SMD	95% CI	N	Weight
**Food−based diet**				
Bendtsen 2018 − high−dairy	0.8	[0.3; 1.3]	22	13.7%
Bendtsen 2018 − low−dairy	0.8	[0.4; 1.2]	30	16.0%
Cotillard 2013 − all	0.2	[−0.1; 0.5]	45	20.3%
Total	0.6	[0.1; 1.0]		50.0%
Heterogeneity: $\chi^2_2 = 8$ (P = .02), $I^2 = 74\%$				
**Roux−en−Y gastric bypass**				
Campisciano 2018 − RYGB	1.1	[0.3; 1.9]	10	7.2%
Chen 2020 − RYGB	0.4	[0.0; 0.9]	20	14.4%
Total	0.7	[0.1; 1.3]		21.6%
Heterogeneity: $\chi^2_1 = 2$ (P = .15), $I^2 = 52\%$				
**Sleeve gastrectomy**				
Chen 2020 − SG	0.4	[0.0; 0.7]	33	18.1%
Campisciano 2018 − SG	0.0	[−0.6; 0.6]	10	10.3%
Total	0.3	[0.0; 0.6]		28.4%
Heterogeneity: $\chi^2_1 = 1$ (P = .33), $I^2 = 0\%$				
Total	0.5	[0.2; 0.7]		100.0%
Heterogeneity: $\chi^2_6 = 13$ (P = .05), $I^2 = 53\%$				
Residual heterogeneity: $\chi^2_4 = 11$ (P = .03), $I^2 = 63\%$				

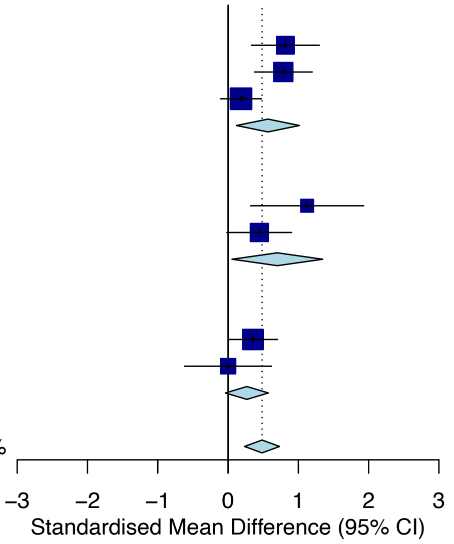

Figure S25: Intestinal permeability changes among studies with lower risk of bias

Source	SMD (95% CI)
**Diet**	
Biolato 2019	0.1 [−0.3; 0.6]
Janczy 2020	−0.1 [−0.7; 0.4]
Total	0.0 [−0.3; 0.4]
Heterogeneity: $\chi^2_1 = 1$ (P = .46), $I^2 = 0\%$	
**Surgery**	
Kellerer 2019	−0.7 [−1.2; −0.1]
Obermayer 2021	−0.7 [−1.3; 0.0]
Clemente−Postigo 2015 −all	−0.4 [−0.7; −0.1]
Monte 2012	−1.1 [−1.7; −0.4]
Troseid 2013	−0.5 [−0.8; −0.2]
Mokhtari 2019	−2.9 [−4.0; −1.9]
vanDielen 2004	−0.3 [−0.7; 0.1]
Total	−0.8 [−1.1; −0.4]
Heterogeneity: $\chi^2_6 = 25$ ($P < .001$), $I^2 = 76\%$	
**Pharmacotherapy**	
Nien 2018 − all	−0.5 [−0.7; −0.3]
Total	−0.5 [−0.7; −0.3]
Heterogeneity: not applicable	
**Diet and surgery**	
Aasbrenn 2020	−1.2 [−1.5; −1.0]
Total	−1.2 [−1.5; −1.0]
Heterogeneity: not applicable	
Total	−0.6 [−0.9; −0.4]
Heterogeneity: $\chi^2_{10} = 60$ ($P < .001$), $I^2 = 83\%$	

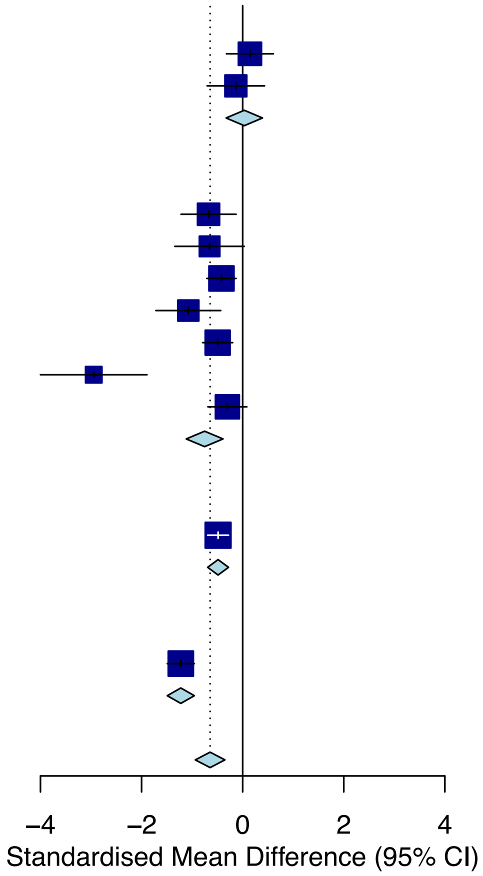

Figure S26: Funnel plots for (A) α-diversity and (B) intestinal permeability.

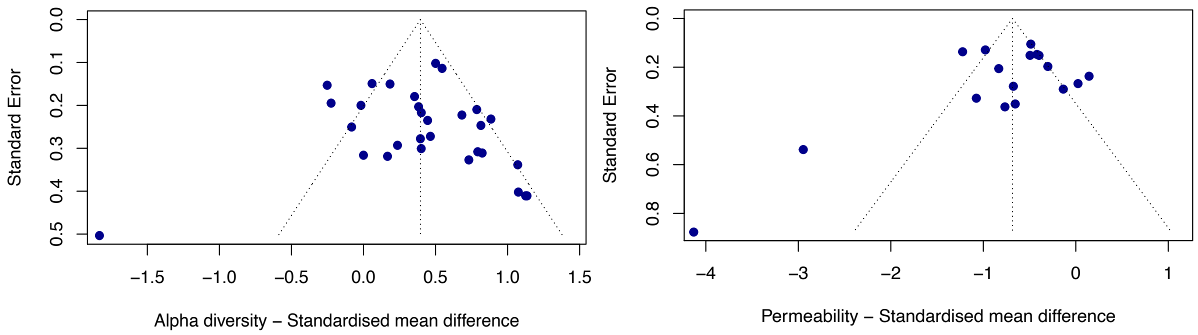

Figure S27: Intestinal permeability changes by intervention excluding the three trials at the left side the funnel plot in Figure S16B.

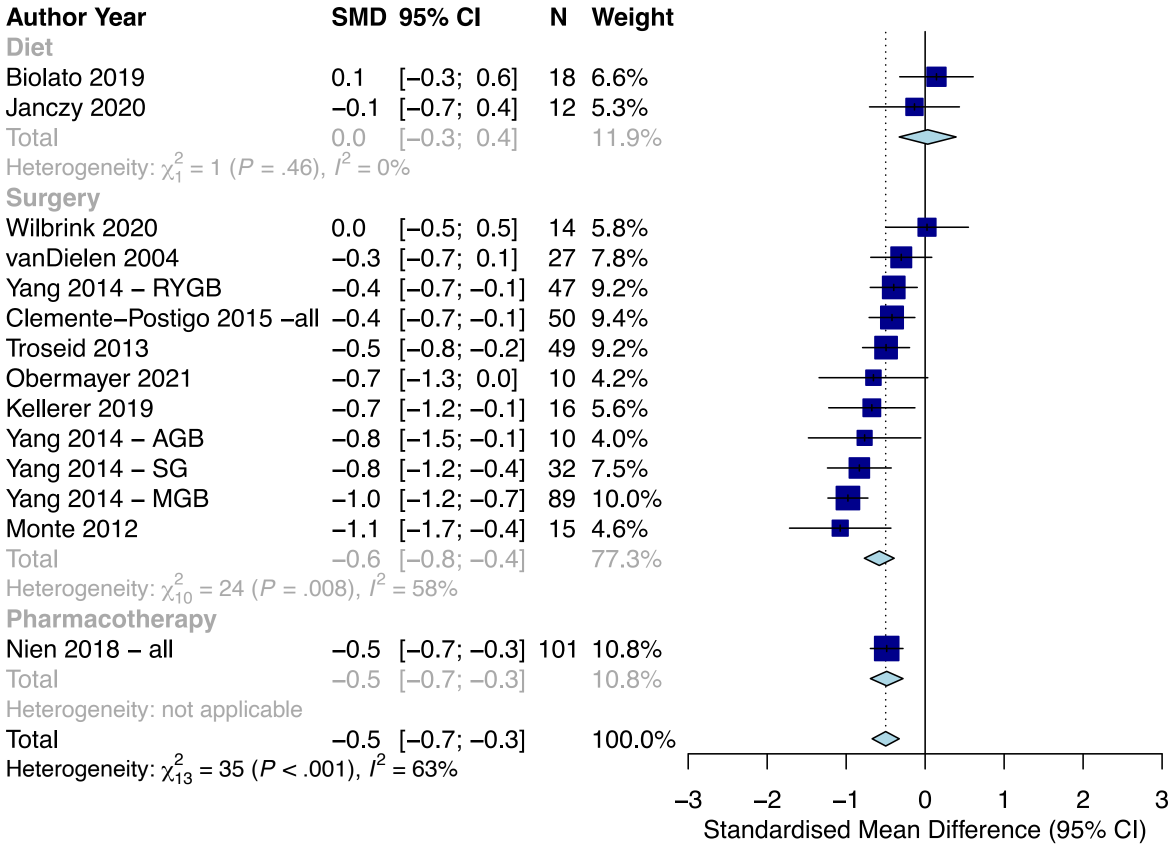

Table S1: Intestinal permeability markers assessed in each study

Study	Marker used in the primary analysis	Additional markers assessed
Wilbrink 2020	Lactulose	Sucrose, sucralose
Obermayer 2021	Lactulose mannitol ratio	
Kellerer 2019	Lactulose mannitol ratio	Plasma serum zonulin, LBP, sucrose, lactulose, mannitol, sucralose
Damms-Machado 2017	Lactulose mannitol ratio	Faecal zonulin, lactulose, mannitol
Monte 2012	Lipopolysaccharide	
Troseid 2013	Lipopolysaccharide	
Clemente-Postigo 2015 -all	Lipopolysaccharide	LBP
Mokhtari 2019	LBP	
Nien 2018 - all	LBP	
vanDielen 2004	LBP	
Yang 2014 - AGB	LBP	
Yang 2014 - MGB	LBP	
Yang 2014 - RYGB	LBP	
Yang 2014 - SG	LBP	
Biolato 2019	^{51}Cr- EDTA	
Aasbrenn 2020	Plasma serum zonulin	
Janczy 2020	Faecal zonulin	

LBP: Lipoprotein binding protein. EDTA: Ethylenediaminetetraacetic acid
Note: In studies where multiple markers were assessed, we used the permeability marker defined as the primary one by the original study authors. Where study authors had not defined the primary marker, we used the most conservative change estimate.

Table S2: Risk of bias assessment

Study	Confounding	Selection	Classification of interventions	Deviations from intended intervention	Missing data	Measurement of outcomes	Selection of reported result	Total score and risk of bias (lower/ higher than the median total score)
Aasbrenn 2020	Moderate	Low	Low	Low	Serious	Low	Low	10, Lower
Al Assal 2020	Low	Low	Low	Low	Serious	Low	Critical	12, Higher
Aron-Wisnewsky 2019	Moderate	Low	Low	Low	Critical	Low	Critical	14, Higher
Bendtsen 2018	Low	Low	Low	Low	Serious	Low	Serious	11, Lower
Biolato 2019	Serious	Low	Low	Low	Low	Low	Low	9, Lower
Blasco 2017	Serious	Low	Low	Low	Low	Low	Moderate	10, Lower
Brinkworth 2009	Low	Low	Low	Low	Moderate	Low	Moderate	9, Lower
Campisciano 2018	Serious	Low	Low	Low	Low	Low	Serious	11, Lower
Chen 2017	Serious	Low	Low	Low	Low	Low	Serious	11, Lower
Chen 2020	Moderate	Low	Low	Low	Critical	Low	Moderate	12, Higher
Clemente-Postigo 2015	Serious	Low	Low	Low	Low	Low	Low	9, Lower
Cotillard 2013	Moderate	Low	Low	Low	Low	Low	Moderate	9, Lower
Damms-Machado 2017	Moderate	Low	Low	Low	Critical	Low	Low	11, Higher
Ejtahed 2018	Low	Low	Low	Moderate	Serious	Low	Serious	12, Higher
Farin 2020	Moderate	Low	Low	Low	Serious	Low	Serious	12, Higher
Frost 2019	Serious	Critical	Moderate	Low	Critical	Low	Critical	19, Higher
Gabel 2020	Serious	Low	Low	Low	Critical	Low	Serious	14, Higher
Henning 2019	Serious	Low	Low	Low	Serious	Low	Serious	13, Higher
Hess 2019	Moderate	Low	Low	Low	Serious	Low	Serious	12, Higher
Janczy 2020	Low	Low	Low	Low	Serious	Low	Serious	11, Lower
Kant 2013	Moderate	Low	Low	Low	Critical	Low	Low	11, Higher
Kellerer 2019	Serious	Low	Low	Low	Low	Moderate	Low	10, Lower
Kikuchi 2018	Serious	Low	Low	Low	Moderate	Moderate	Serious	13, Higher
Kong 2013	Moderate	Moderate	Low	Low	Critical	Low	Critical	15, Higher
Lin 2019	Serious	Critical	Low	Low	Moderate	Low	Critical	16, Higher
Liu 2017	Serious	Low	Low	Low	Low	Low	Critical	12, Higher
Louis 2016	Low	Serious	Low	Low	Low	Low	Critical	12, Higher
Medina-Vera 2019	Moderate	Low	Low	Low	Critical	Low	Critical	14, Higher
Mokhtari 2019	Low	Low	Low	Low	Moderate	Low	Low	8, Lower
Monte 2012	Serious	Low	Low	Low	Low	Low	Low	9, Lower
MunizPedrogo 2018	Low	Low	Low	Low	Critical	Low	Critical	13, Higher
Murphy 2017	Serious	Critical	Low	Low	Low	Low	Critical	15, Higher
Nien 2018	Serious	Moderate	Moderate	Low	Low	Low	Low	11, Lower
Obermayer 2021	Serious	Low	Low	Low	Low	Low	Low	9, Lower
Paganelli 2019	Critical	Low	Low	Low	Low	Low	Moderate	11, Higher
Palleja 2016	Serious	Serious	Low	Low	Low	Low	Moderate	12, Higher
Palmisano 2019	Moderate	Low	Low	Low	Low	Low	Moderate	9, Lower

Study	Confounding	Selection	Classification of interventions	Deviations from intended intervention	Missing data	Measurement of outcomes	Selection of reported result	Total score and risk of bias (lower/ higher than the median total score)
Patrone 2016	Serious	Low	Low	Low	Low	Low	Serious	11, Lower
Remely 2015	Low	Serious	Low	Low	Critical	Low	Critical	15, Higher
Sanchez 2014	Serious	Low	Low	Low	Serious	Low	Critical	14, Higher
Sanchez-Alcoholado 2019	Critical	Moderate	Low	Low	Low	Low	Serious	13, Higher
Shen 2019	Low	Low	Low	Low	Low	Low	Critical	10, Higher
Simoes 2014	Serious	Low	Low	Low	Low	Low	Serious	11, Lower
Troseid 2013	Serious	Low	Low	Low	Low	Low	Low	9, Lower
vanDielen 2004	Moderate	Low	Low	Low	Low	Low	Low	8, Lower
Wilbrink 2020	Moderate	Moderate	Low	Low	Critical	Low	Critical	15, Higher
Yang 2014	Critical	Low	Low	Low	Low	Low	Low	10, Higher

Search strategy

Medline

	Searches
1	Weight Loss/
2	Weight Reduction Programs/
3	diet therapy/ or caloric restriction/ or diet, reducing/
4	exp Obesity/dh [Diet Therapy]
5	(weight adj3 (loss or lose or lost or losing or chang* or reduc* or manag*)).ti,ab,kw.
6	((weight or overweight or obes*) adj5 (program* or service? or intervention?)).ti,ab,kw.
7	exp Anti-Obesity Agents/
8	exp Obesity/dt, th
9	((weight or overweight or obes*) adj3 (therap* or treat* or drug? or agent?)).ti,ab.
10	exp OBESITY/su
11	exp Bariatric Surgery/
12	((weight loss or bariatric or obes*) adj5 surg*).ti,ab. or bariatric*.ti.
13	(((gastric or jejunoileal) adj3 (band* or bypass* or balloon* or diver*)) or gastrectom* or gastroplast* or ((biliopancreatic or bilio-pancreatic) adj2 diver*)).ti,ab,kw.
14	obesity management/ or bariatrics/
15	((obes* or overweight) adj3 manage*).ti,ab,kw.
16	1 or 2 or 3 or 4 or 5 or 6 or 7 or 8 or 9 or 10 or 11 or 12 or 13 or 14 or 15
17	exp Microbiota/
18	(exp Intestines/ or Intestinal Mucosa/) and (Permeability/ or Inflammation/)
19	exp *Fatty Acids, Volatile/
20	(microbiome or microbiota or biome or biota).ti,ab,kw.
21	((gut or gastro* or gastric or intestin* or faec* or fec*) adj3 (organism? or microorganism? or microbe? or microflora or bacteria)).ti,ab,kw.
22	((intestin* or gut) adj2 (permeab* or inflamm*)).ti,ab,kw.
23	((short chain or volatile) adj fatty acid?).ti,ab,kw.
24	((alpha or a or beta or b) adj2 divers*).ti,ab,kw.
25	(Bray curtis adj3 (distance or dissimilar* or index)).ti,ab,kw.
26	(shannon adj3 (distance or dissimilar* or index)).ti,ab,kw.
27	((otu or otus or operational taxonomic unit?) adj5 (count* or sequenc* or table? or index)).ti,ab,kw.
28	chao1.ti,ab,kw.
29	(species adj3 (divers* or dissimilar* or abundance)).ti,ab,kw.

30	17 or 18 or 19 or 20 or 21 or 22 or 23 or 24 or 25 or 26 or 27 or 28 or 29
31	randomized controlled trial.pt.
32	controlled clinical trial.pt.
33	randomized.ab.
34	placebo.ab.
35	drug therapy.fs.
36	randomly.ab.
37	trial.ab.
38	groups.ab.
39	prospective studies/ or controlled before-after studies/ or interrupted time series analysis/ or pilot projects/
40	clinical trial/
41	(intervention? or preintervention? or postintervention?).ti,ab.
42	((pilot or feasibility) adj2 (stud* or project*)).ti,ab.
43	(prospective adj2 (stud* or project*)).ti,ab.
44	(single arm adj2 (stud* or project*)).ti,ab.
45	(before adj2 after).ti,ab.
46	(interrupted time series or timepoint? or endpoint?).ti,ab.
47	31 or 32 or 33 or 34 or 35 or 36 or 37 or 38 or 39 or 40 or 41 or 42 or 43 or 44 or 45 or 46
48	exp animals/ not humans.sh.
49	(rat or rats or rodent? or mice or mouse or murine).ti.
50	48 or 49
51	47 not 50
52	16 and 30 and 51

Embase

	Searches
1	weight reduction/
2	weight loss program/
3	diet therapy/ or exp diet restriction/ or low calory diet/ or low fat diet/
4	exp Obesity/dm
5	(weight adj3 (loss or lose or lost or losing or chang* or reduc* or manag*)).ti,ab,kw.
6	((weight or overweight or obes*) adj5 (program* or service? or intervention?)).ti,ab,kw.
7	exp antiobesity agent/
8	exp Obesity/dt, th
9	((weight or overweight or obes*) adj3 (therap* or treat* or drug? or agent?)).ti,ab.
10	exp OBESITY/su
11	exp Bariatric Surgery/
12	((weight loss or bariatric or obes*) adj5 surg*).ti,ab. or bariatric*.ti.
13	(((gastric or jejunoileal) adj3 (band* or bypass* or balloon* or diver*)) or gastrectom* or gastroplast* or ((biliopancreatic or bilio-pancreatic) adj2 diver*)).ti,ab,kw.
14	obesity management/ or bariatrics/
15	((obes* or overweight) adj3 manage*).ti,ab,kw.
16	1 or 2 or 3 or 4 or 5 or 6 or 7 or 8 or 9 or 10 or 11 or 12 or 13 or 14 or 15
17	*microflora/ or exp *intestine flora/ or exp microbiome/
18	intestine mucosa permeability/
19	exp *short chain fatty acid/
20	(microbiome or microbiota or biome or biota).ti,ab,kw.
21	((gut or gastro* or gastric or intestin* or faec* or fec*) adj3 (organism? or microorganism? or microbe? or microflora or bacteria)).ti,ab,kw.
22	((intestin* or gut) adj2 (permeab* or inflamm*)).ti,ab,kw.
23	((short chain or volatile) adj fatty acid?).ti,ab,kw.
24	((alpha or a or beta or b) adj2 divers*).ti,ab,kw.
25	(Bray curtis adj3 (distance or dissimilar* or index)).ti,ab,kw.
26	(shannon adj3 (distance or dissimilar* or index)).ti,ab,kw.
27	((otu or otus or operational taxonomic unit?) adj5 (count* or sequenc* or table? or index)).ti,ab,kw.
28	chao1.ti,ab,kw.
29	(species adj3 (divers* or dissimilar* or abundance)).ti,ab,kw.
30	17 or 18 or 19 or 20 or 21 or 22 or 23 or 24 or 25 or 26 or 27 or 28 or 29
31	randomized controlled trial/
32	single blind procedure/ or double blind procedure/
33	crossover procedure/
34	(random* or ((singl* or doubl*) adj (blind* or mask*)) or crossover or cross over or factorial* or latin square or assign* or allocat* or volunteer*).ti,ab.

35	intervention study/ or prospective study/ or feasibility study/ or pilot study/
36	clinical trial/
37	(intervention? or preintervention? or postintervention?).ti,ab.
38	((pilot or feasibility) adj2 (stud* or project*)).ti,ab.
39	(prospective adj2 (stud* or project*)).ti,ab.
40	(single arm adj2 (stud* or project*)).ti,ab.
41	(before adj2 after).ti,ab.
42	(interrupted time series or timepoint? or endpoint?).ti,ab.
43	31 or 32 or 33 or 34 or 35 or 36 or 37 or 38 or 39 or 40 or 41 or 42
44	(exp animals/ or nonhuman/) not human/
45	(rat or rats or rodent? or mice or mouse or murine).ti.
46	44 or 45
47	43 not 46
48	16 and 30 and 47

CINAHL

	Query
S1	(MH "Microbiota+")
S2	TX (microbiome or microbiota or biome or biota) OR TX ((gut or gastro* or gastric or intestin* or faec* or fec*) N3 (organism* or microorganism* or microbe* or microflora or bacteria)) OR TX ((intestin* or gut) N2 (permeab* or inflamm*)) OR TX ("short chain fatty acid*" or "volatile fatty acid*") OR TX (((alpha or beta) N2 divers*)) OR TX (("Bray curtis" N3 (distance or dissimilar* or index))) OR TX ((shannon N3 (distance or dissimilar* or index))) OR TX (((otu or otus or "operational taxonomic unit*") N5 (count* or sequenc* or table* or index))) OR TX chao1 OR TX ((species N3 (divers* or dissimilar* or abundance)))
S3	S1 OR S2
S4	(MH "Weight Loss") OR (MH "Weight Reduction Programs")
S5	(MH "Diet, Reducing") OR (MH "Diet Therapy")
S6	TI ((weight N3 (loss or lose or lost or losing or chang* or reduc* or manag*))) OR AB ((weight N3 (loss or lose or lost or losing or chang* or reduc* or manag*))) OR TI (((energy or calori*) N2 (reduc* or restrict))) OR AB (((energy or calori*) N2 (reduc* or restrict))) OR TI (((weight or overweight or obes*) N5 (program* or service? or intervention?))) OR AB (((weight or overweight or obes*) N5 (program* or service? or intervention?)))
S7	(MH "Antiobesity Agents+")
S8	TI (((weight or overweight or obes*) N3 (therap* or treat* or drug? or agent?))) AND AB (((weight or overweight or obes*) N3 (therap* or treat* or drug? or agent?)))
S9	(MH "Bariatric Surgery+")
S10	TI (((weight loss or bariatric or obes*) N5 surg*)) OR AB (((weight loss or bariatric or obes*) N5 surg*)) OR TI bariatric* OR TI ((((gastric or jejunoileal) N3 (band* or bypass* or balloon* or diver*)) or gastrectom* or gastroplast* or ((biliopancreatic or bilio-pancreatic) N2 diver*))) OR AB ((((gastric or jejunoileal) N3 (band* or bypass* or balloon* or diver*)) or gastrectom* or gastroplast* or ((biliopancreatic or bilio-pancreatic) N2 diver*)))
S11	(MH "Obesity+/DH/DT/SU/TH")
S12	S4 OR S5 OR S6 OR S7 OR S8 OR S9 OR S10 OR S11
S13	S3 AND S12
S14	S3 AND S12 Limiters - Clinical Queries: Therapy - Best Balance
S15	(MH "Prospective Studies")
S16	TI (intervention? or preintervention? or postintervention?) OR AB (intervention? or preintervention? or postintervention?)
S17	TI (((pilot or feasibility) N2 (stud* or project*))) OR AB (((pilot or feasibility) N2 (stud* or project*)) or postintervention?)
S18	TI ((prospective N2 (stud* or project*))) OR AB ((prospective N2 (stud* or project*)))

S19 TI ((single arm N2 (stud* or project*))) OR AB ((single arm N2 (stud* or project*)))

S20 TI (before N2 after) OR AB (before N2 after)

S21 TI ((interrupted time series or timepoint? or endpoint?)) OR AB ((interrupted time series or timepoint? or endpoint?))

S22 S15 OR S16 OR S17 OR S18 OR S19 OR S20 OR S21

S23 S3 AND S12 AND S22

S24 S14 OR S23

Cochrane

ID	Search
#1	weight or obes* or overweight or bariatric*:ti,ab,kw (Word variations have been searched)
#2	MeSH descriptor: [Weight Loss] this term only
#3	MeSH descriptor: [Weight Reduction Programs] explode all trees
#4	MeSH descriptor: [Diet Therapy] this term only
#5	MeSH descriptor: [Diet, Reducing] explode all trees
#6	MeSH descriptor: [Caloric Restriction] explode all trees
#7	((weight near/3 (loss or lose or lost or losing or chang* or reduc* or manag*))):ti,ab,kw OR (((energy or calori*) near/2 (reduc* or restrict))):ti,ab,kw OR (((weight or overweight or obes*) near/3 (program* or service* or intervention*))):ti,ab,kw (Word variations have been searched)
#8	MeSH descriptor: [Anti-Obesity Agents] explode all trees
#9	(((weight or overweight or obes*) near/3 (therap* or treat* or drug* or agent*))):ti,ab,kw (Word variations have been searched)
#10	MeSH descriptor: [Bariatric Surgery] explode all trees
#11	(bariatric*):ti OR ((("weight loss" or bariatric or obes*) near/3 surg*)):ti,ab,kw OR (((gastric or jejunoileal) near/3 (band* or bypass* or balloon* or diver*)) or gastrectom* or gastroplast*):ti,ab,kw OR (((biliopancreatic or bilio-pancreatic) near/2 diver*)):ti,ab,kw OR (((obes* or overweight) near/3 manage*)):ti,ab,kw (Word variations have been searched)
#12	MeSH descriptor: [Obesity] explode all trees
#13	MeSH descriptor: [Obesity Management] explode all trees
#14	#1 or #2 or #3 or #4 or #5 or #6 or #7 or #8 or #9 or #10 or #11 or #12 or #13
#15	MeSH descriptor: [Microbiota] explode all trees
#16	MeSH descriptor: [Fatty Acids, Volatile] explode all trees
#17	((microbiome or microbiota or biome or biota)):ti,ab,kw OR (((gut or gastro* or gastric or intestin* or faec* or fec*) NEAR/3 (organism* or microorganism* or microbe* or microflora or bacteria))):ti,ab,kw OR (((intestin* or gut) NEAR/2 (permeab* or inflamm*))):ti,ab,kw
#18	(((short chain or volatile) NEXT "fatty acid*")):ti,ab,kw
#19	(((alpha or beta) NEAR/2 divers*)):ti,ab,kw OR (("Bray curtis" NEAR/3 (distance or dissimilar* or index))):ti,ab,kw OR ((shannon NEAR/3 (distance or dissimilar* or index))):ti,ab,kw OR (((otu or otus or "operational taxonomic unit*") NEAR/5 (count* or sequenc* or table* or index))):ti,ab,kw OR (chao1):ti,ab,kw
#20	((species NEAR/3 (divers* or dissimilar* or abundance))):ti,ab,kw
#21	#15 or #16 or #17 or #18 or #19 or #20
#22	#14 and #21

Clinicaltrials.gov

Other terms=(microbiome OR microbiota OR biota OR biome OR "gut permeability" OR "intestinal permeability" OR "gut inflammation" OR "intestinal inflammation") AND Condition=(obesity OR obesity OR weight OR overweight OR bariatric OR bariatrics)

Other terms=("short chain fatty acids" OR "volatile fatty acids") AND Condition=(obesity OR obesity OR weight OR overweight OR bariatric OR bariatrics)

Other terms=("species diversity" OR "diverse species" OR "species dissimilarity" OR "species dissimilarities" OR "species adundance") AND Condition=(obesity OR obesity OR weight OR overweight OR bariatric OR bariatrics)
